# Supplementary material for: The Residual Efficacy of SumiShield™ 50WG and K-Othrine® WG250 IRS Formulations Applied to Different Building Materials against Anopheles and Aedes Mosquitoes
Source: Insects. 2022 Jan 20;13(2):112. doi: 10.3390/insects13020112 (PMC8877416; doi:10.3390/insects13020112)
Supplement: Supplementary file 1 [file insects-13-00112-s001.zip › insects-1516983-SI/Supplementary Material/Supplementary Material_The residual efficacy of SumiShield 50WG and K-Othrine WG250 IRS formulations.pdf]

Supplementary Material

The residual efficacy of SumiShield™ 50WG and K-Othrine® WG250 IRS formulations applied to different building materials against *Anopheles* and *Aedes* mosquitoes

Rosemary Susan Lees[1*], Giorgio Praulins[1], Natalie Lissenden[1], Andy South[1], Jessie Carson[1], Faye Brown[1], John Lucas[2], Dave Malone[3]

Table S1. The physical characteristics and chemical properties of the mud used to prepare the mud surfaces used for testing. Characterisation was conducted by ACS Testing Ltd (Poole, Dorset) to determine the physical characteristics and chemical properties of the mud.

Property	Sample A	Sample B
**Elemental Analysis (% by weight)**		
Aluminium	4	3.58
Calcium	0.23	0.12
Iron	1.5	1.24
Potassium	0.14	0.13
Magnesium	0.07	0.06
Sodium	0.004	0.004
Titanium	0.02	1.6
Arsenic	<0.001	Nil
Cadmium	Nil	Nil
Chromium	0.0052	0.0047
Copper	0.0017	0.0016
Mercury	<0.0001	<00001
Nickel	0.0028	0.0026
Lead	0.0005	0.0005
Selenium	0.0008	0.0003
Zinc	0.0017	0.0015
Manganese	0.0066	0.0052
Phosphorus	0.012	0.011
Sulphur	0.0068	0.0054
Vanadium	0.0041	0.0035
Barium	0.0042	0.0041
Silica	ND	ND
**Properties**		
Organic matter (%)	1.43	0.0187
Acid soluble sulphate (%)	0.03	0.02
CEC (cation exchange capacity)	21.1	13.8
Acid soluble chloride	<0.01	<0.01
pH	7.1	5.9
Water soluble chloride	6.01	5.97
Water soluble fluoride (mg/l)	0.13	0.06

Water soluble phosphate (mg/l)	<0.02	<0.02
Water soluble sulphate	6.34	3.2
Water soluble nitrate	1.75	1.79
**Physical Properties**		
Optimum moisture content (%)*	17	17
Dry density (mg/m3)	1.52	ND
Plastic limit (%) ^	12	13
Liquid limit (%) $	30	30
% passing 425 microns	84	74
Porosity calculated TW (% voids)	43	ND
**Composition**		
Cobbles (60-200mm) (%)	0	0
Gravel (2-60mm) (%)	24	28
Sand (63microns-2mm) (%)	28	35
Silt (2-63microns) (%)	25	19
Clay (<2microns) (%)	23	18

* The water content at which a maximum dry unit weight can be achieved after a given compaction effort.

^ Determined by rolling out a thread of the fine portion of a soil on a flat, non-porous surface.

$ The water content at which the behavior of a clayey soil changes from the plastic state to the liquid state.

Table S2. Intrinsic activity of clothianidin, measured as mortality after topical application in solution with acetone, in susceptible strains of *Anopheles gambiae* (Kisumu) and *Aedes aegypti* (New Orleans). Kisumu data has been previously published [24]. Mean mortality represents an average of three cohorts of ten 2-5 day old non-blood fed female adult mosquitoes, corrected for negative control mortality using Abbott's formula [23].

**Property**	***An. gambiae* Kisumu**		***Ae. aegypti* New Orleans**	
**Concentration (%)**	**Mean mortality 24 hours (%)**	**Mean mortality 48 hours (%)**	**Mean mortality 24 hours (%)**	**Mean mortality 48 hours (%)**
0.01	100	100	100	100
0.1	97	100	97	100
1	97	97	97	97
Acetone control	6	12	7	13
0.1% permethrin	100	100	100	100

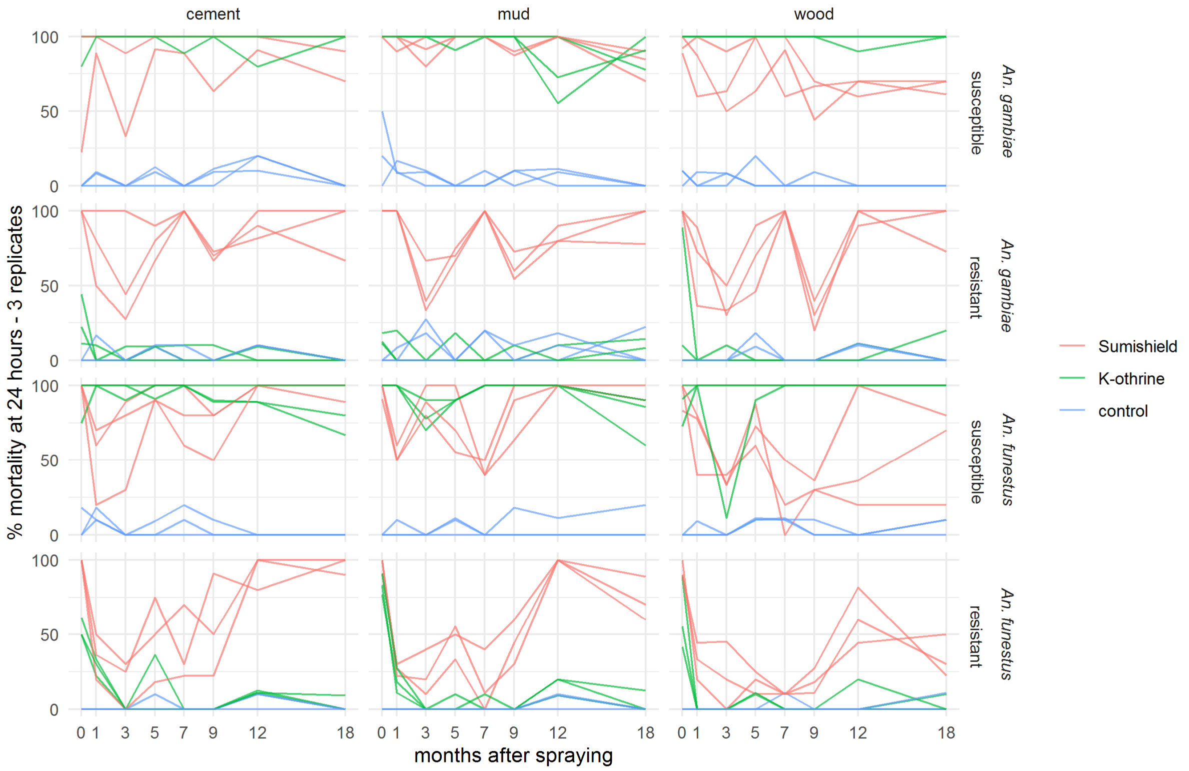

Figure S1. Residual efficacy of SumiShield and K-Othrine applied to different surface types against *Anopheles* mosquitoes. Mortality of resistant and susceptible strains of *An. gambiae* and *An. funestus* at 24-hours, after exposure to cement, mud and wood surfaces treated with SumiShield or K-Othrine is presented, in comparison to control surfaces treated with water only. Mosquitoes were exposed in a WHO cone bioassay at 24-hours, and 1, 3, 5, 7, 9, 12, and 18 months after surfaces were treated. Data from 3 replicates of each treatment and surface type are presented as separate lines.

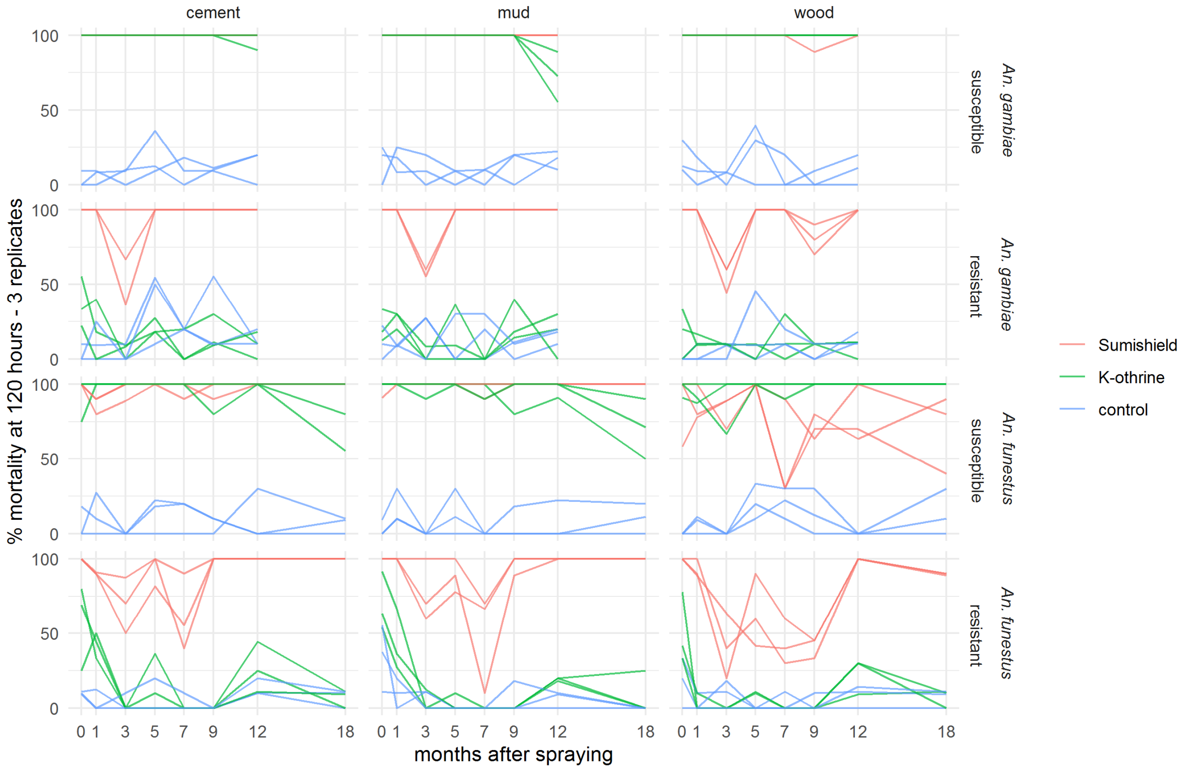

Figure S2. Residual efficacy of SumiShield and K-Othrine applied to different surface types against *Anopheles* mosquitoes. Mortality of resistant and susceptible strains of *An. gambiae* and *An. funestus* at 120-hours, after exposure to cement, mud and wood surfaces treated with SumiShield or K-Othrine is presented, in comparison to control surfaces treated with water only. Mosquitoes were exposed in a WHO cone bioassay at 24-hours, and 1, 3, 5, 7, 9, 12, and 18 months after surfaces were treated. Data from 3 replicates of each treatment and surface type are presented as separate lines.

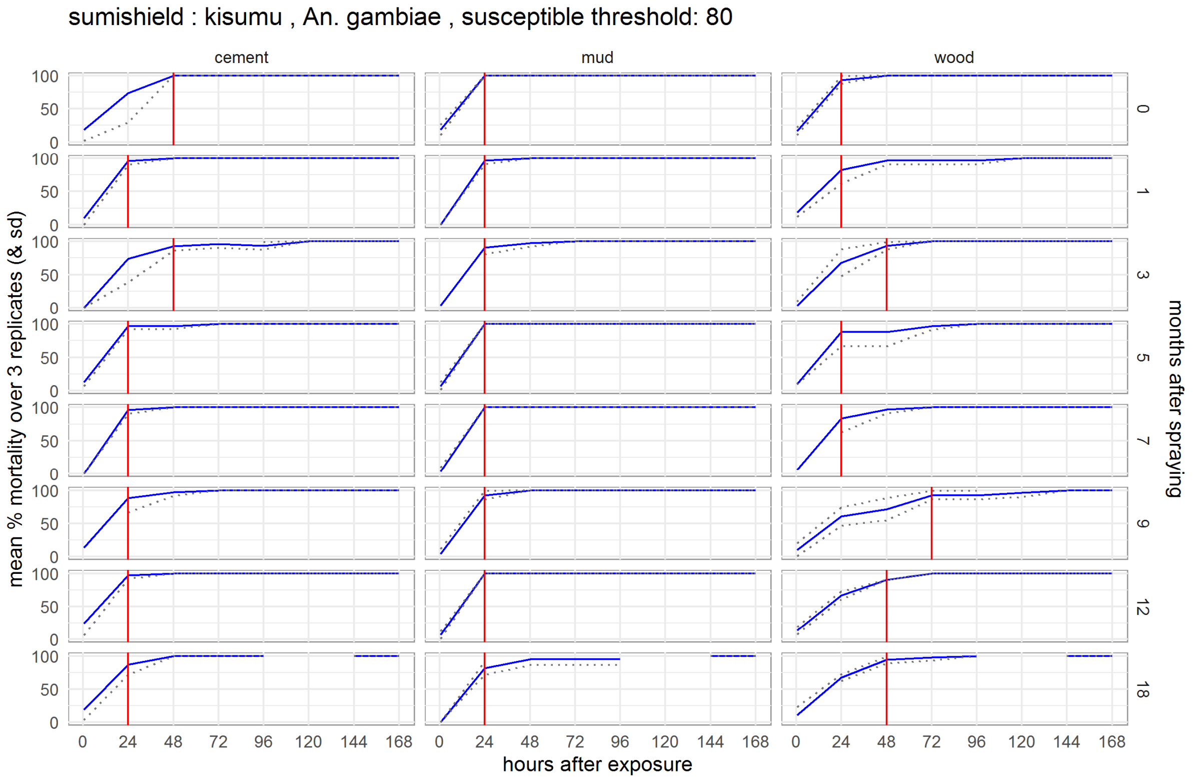

Figure S3. Speed of action of SumiShield against insecticide-susceptible *An. Gambiae* Kisumu. Cumulative mortality is shown by hours post-exposure to treated cement, mud, and wood surfaces, for each month of the experiment. Time to reach the WHO-recommended 80% mortality threshold for an IRS product is marked with a red line.

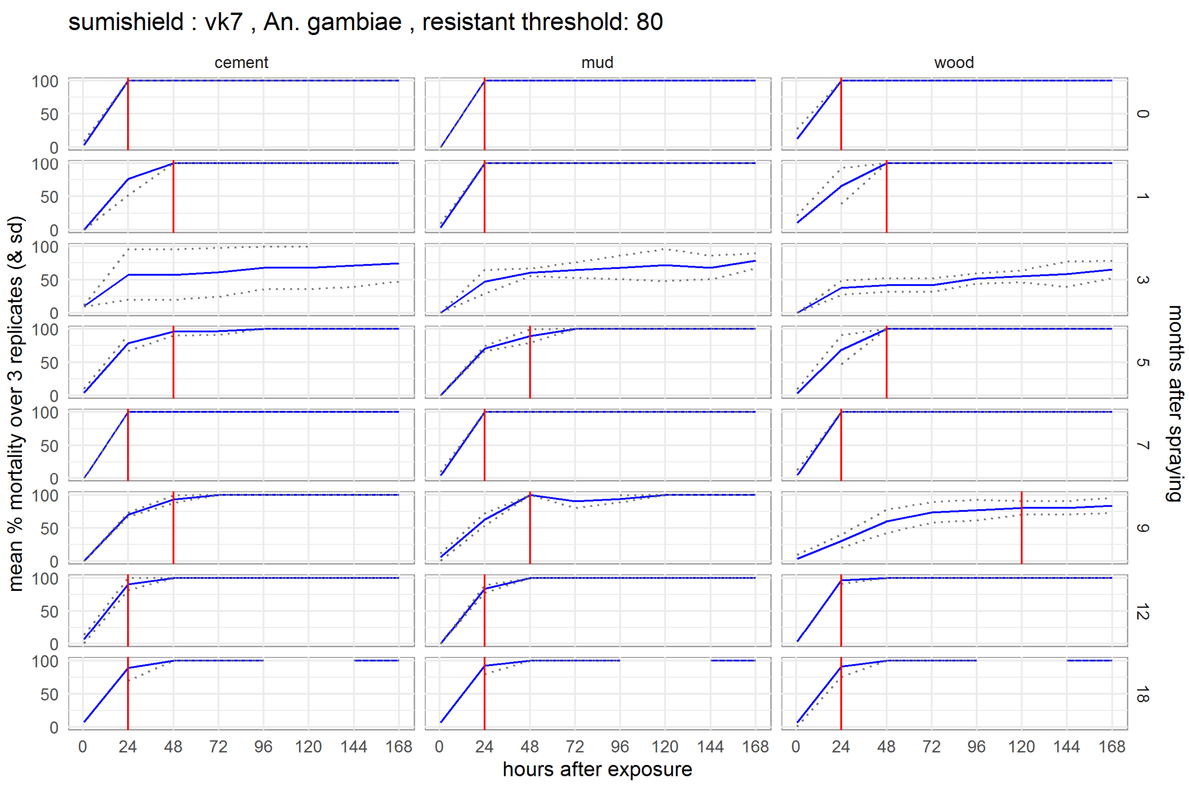

Figure S4. Speed of action of SumiShield against insecticide-resistant *An. Gambiae* VK7. Cumulative mortality is shown by hours post-exposure to treated cement, mud, and wood surfaces, for each month of the experiment. Time to reach the WHO-recommended 80% mortality threshold for an IRS product is marked with a red line.

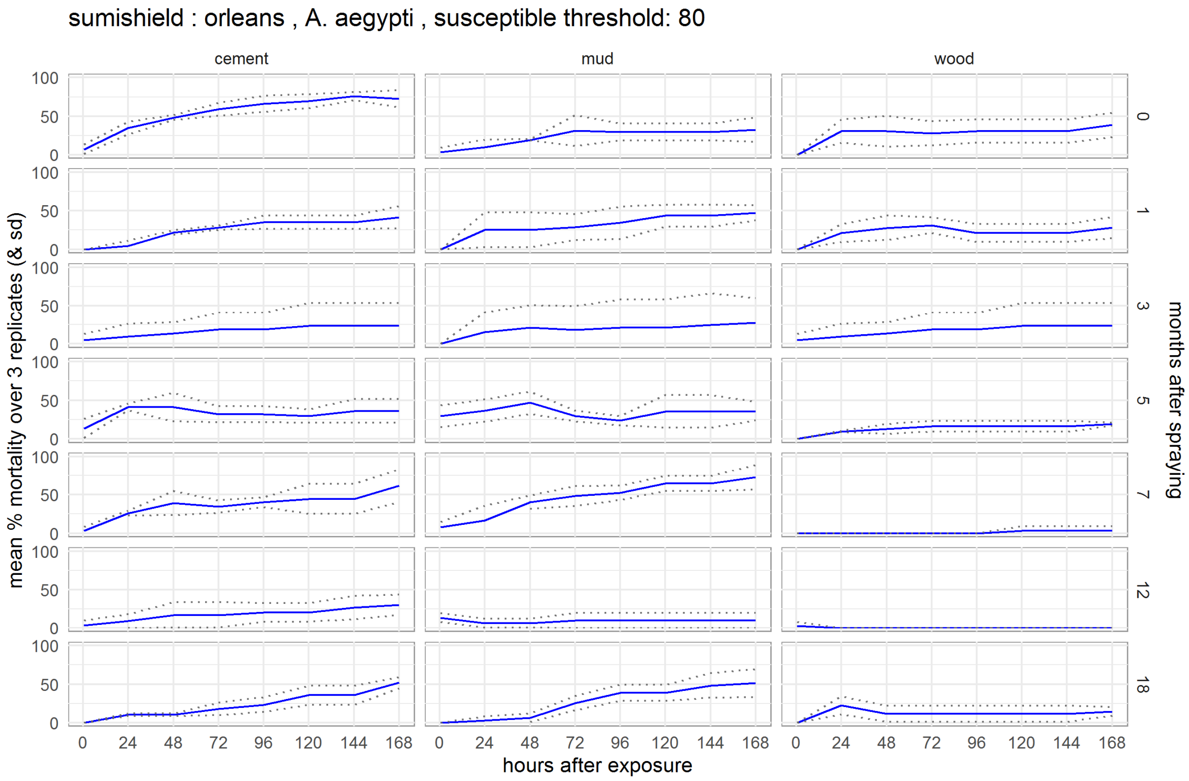

Figure S5. Speed of action of SumiShield against insecticide-susceptible *Ae. Aegypti* New Orleans. Cumulative mortality is shown by hours post-exposure to treated cement, mud, and wood surfaces, for each month of the experiment. Time to reach the WHO-recommended 80% mortality threshold for an IRS product is marked with a red line.

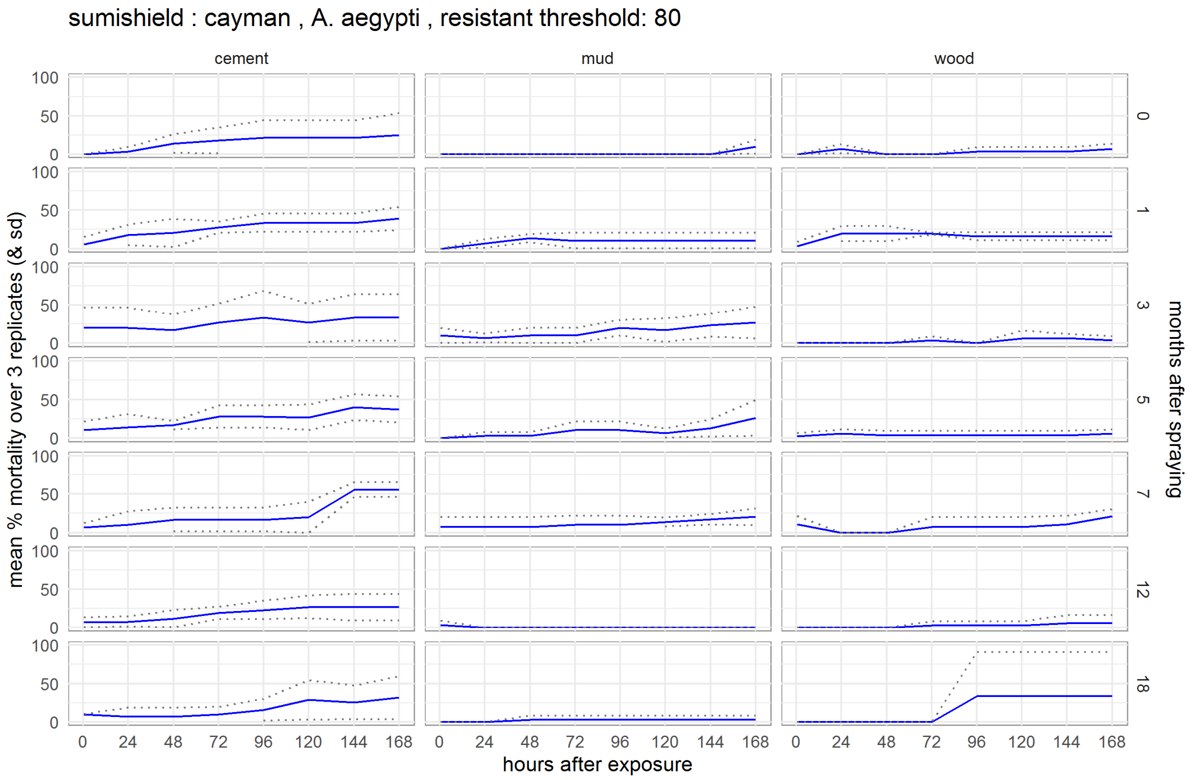

Figure S6. Speed of action of SumiShield against insecticide-resistant *Ae. Aegypti* Cayman. Cumulative mortality is shown by hours post-exposure to treated cement, mud, and wood surfaces, for each month of the experiment. Time to reach the WHO-recommended 80% mortality threshold for an IRS product is marked with a red line.

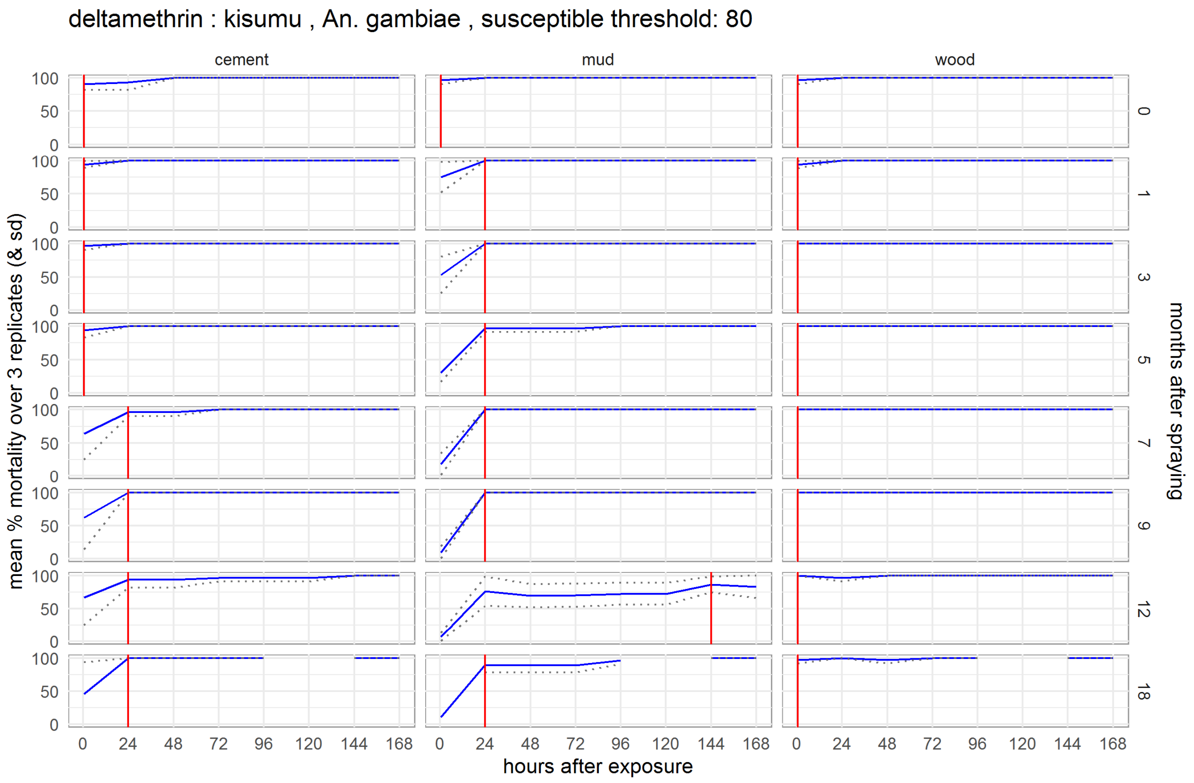

Figure S7. Speed of action of K-Othrine (deltamethrin) against insecticide-susceptible *An. gambiae* Kisumu. Cumulative mortality is shown by hours post-exposure to treated cement, mud, and wood surfaces, for each

month of the experiment. Time to reach the WHO-recommended 80% mortality threshold for an IRS product is marked with a red line.

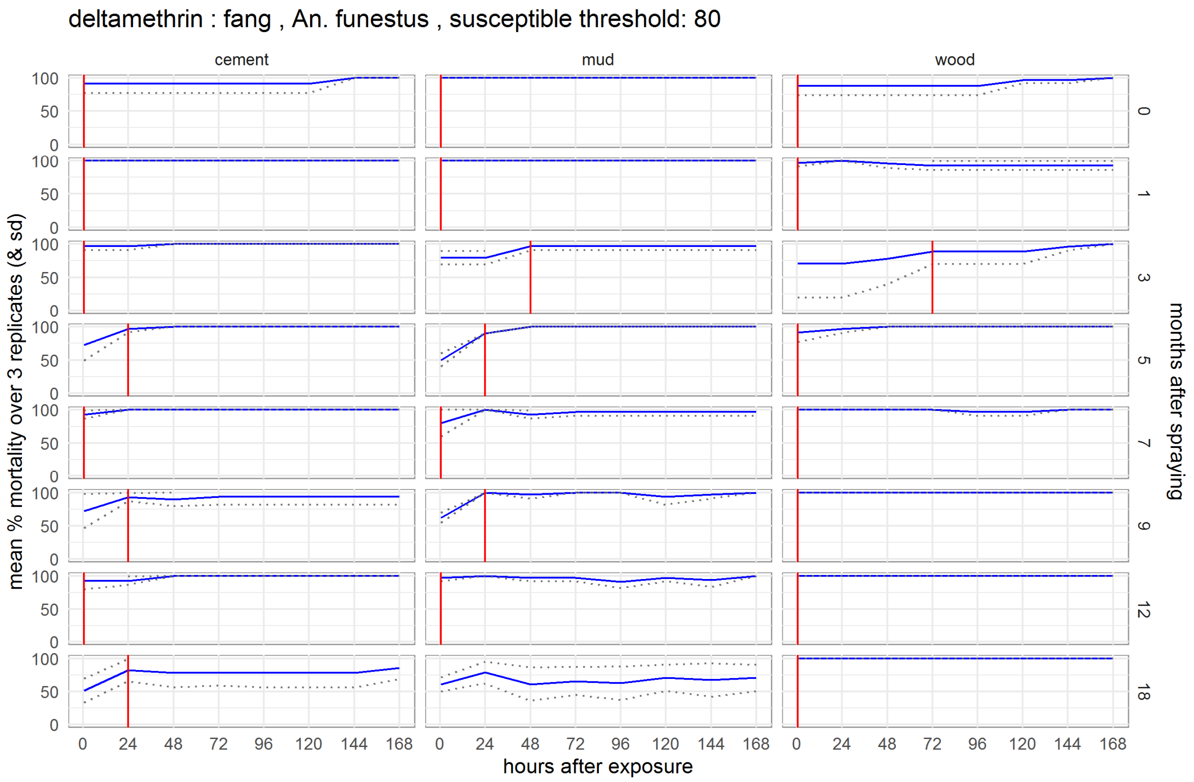

Figure S8. Speed of action of K-Othrine (deltamethrin) against insecticide-susceptible *An. funestus* Fang. Cumulative mortality is shown by hours post-exposure to treated cement, mud, and wood surfaces, for each month of the experiment. Time to reach the WHO-recommended 80% mortality threshold for an IRS product is marked with a red line.

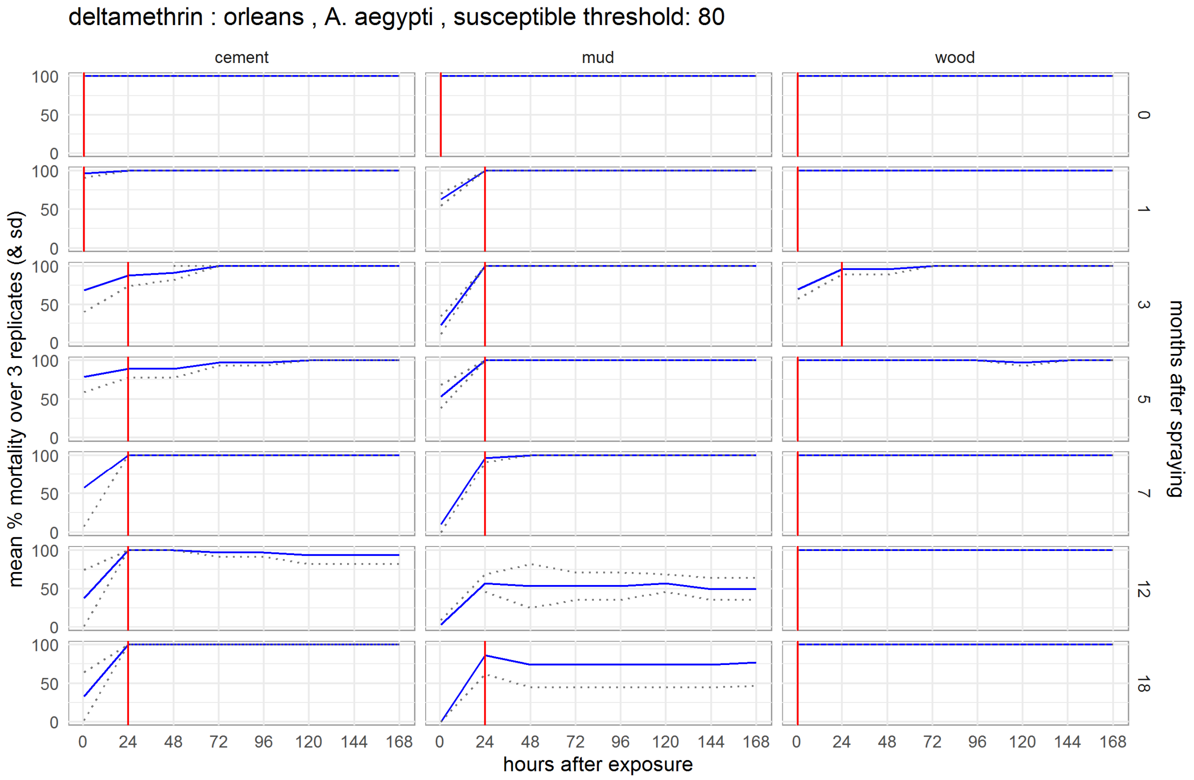

Figure S9. Speed of action of K-Othrine (deltamethrin) against insecticide-susceptible *Ae. aegypti* New Orleans. Cumulative mortality is shown by hours post-exposure to treated cement, mud, and wood surfaces, for each month of the experiment. Time to reach the WHO-recommended 80% mortality threshold for an IRS product is marked with a red line.

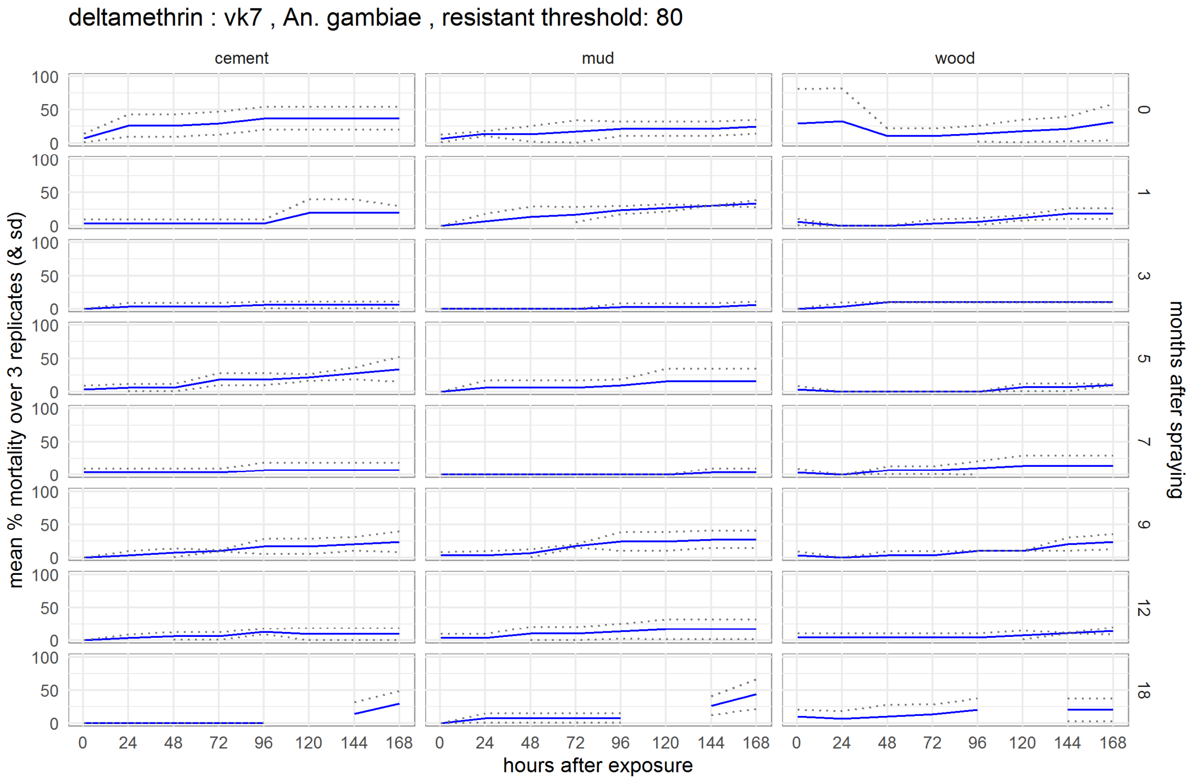

Figure S10. Speed of action of K-Othrine (deltamethrin) against insecticide-resistant *An. gambiae* VK7 2014. Cumulative mortality is shown by hours post-exposure to treated cement, mud, and wood surfaces, for each

month of the experiment. Time to reach the WHO-recommended 80% mortality threshold for an IRS product is marked with a red line.

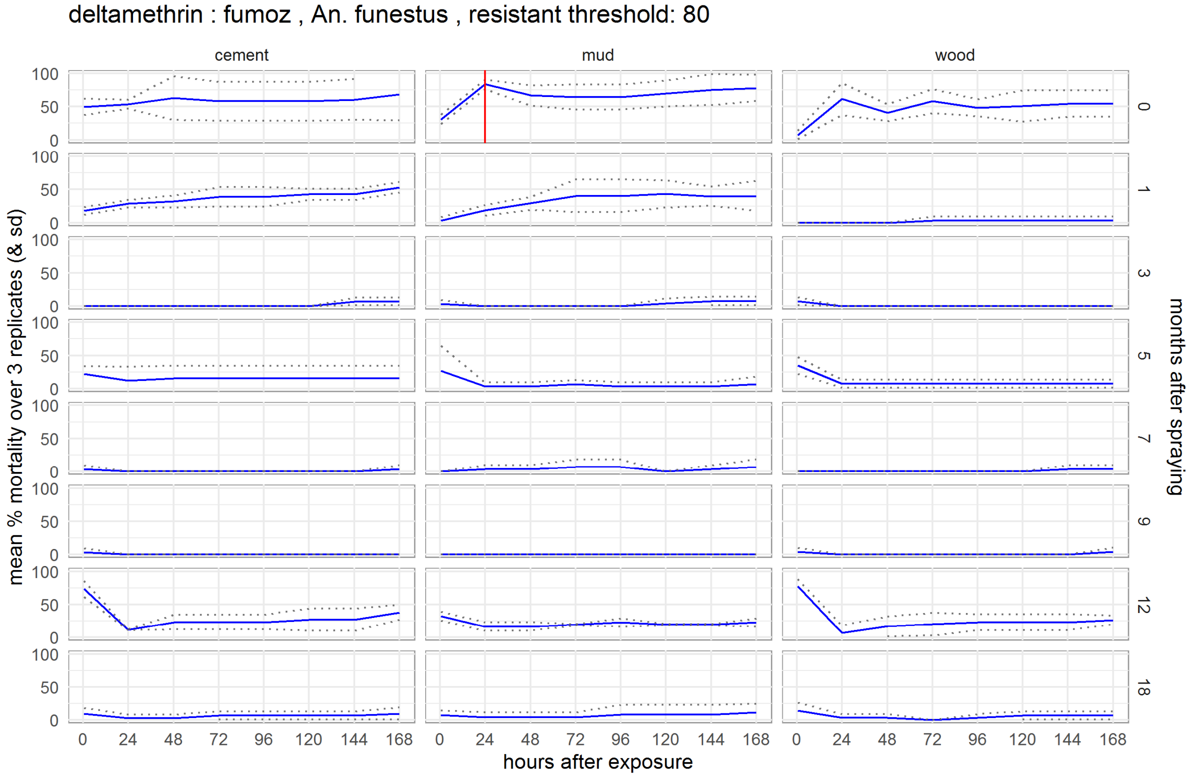

Figure S11. Speed of action of K-Othrine (deltamethrin) against insecticide-resistant *An. funestus* FUMOZ-R. Cumulative mortality is shown by hours post-exposure to treated cement, mud, and wood surfaces, for each month of the experiment. Time to reach the WHO-recommended 80% mortality threshold for an IRS product is marked with a red line.

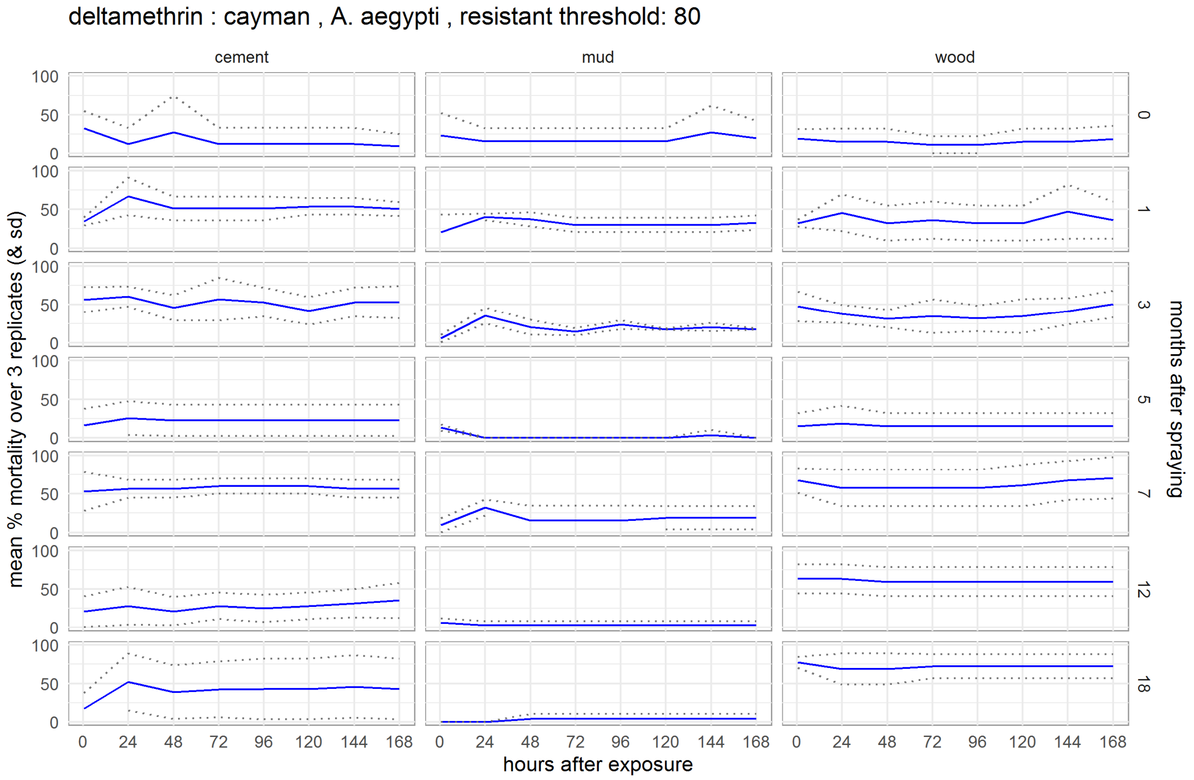

Figure S12. Speed of action of K-Othrine (deltamethrin) against insecticide-resistant *Ae. aegypti* Cayman. Cumulative mortality is shown by hours post-exposure to treated cement, mud, and wood surfaces, for each month of the experiment. Time to reach the WHO-recommended 80% mortality threshold for an IRS product is marked with a red line

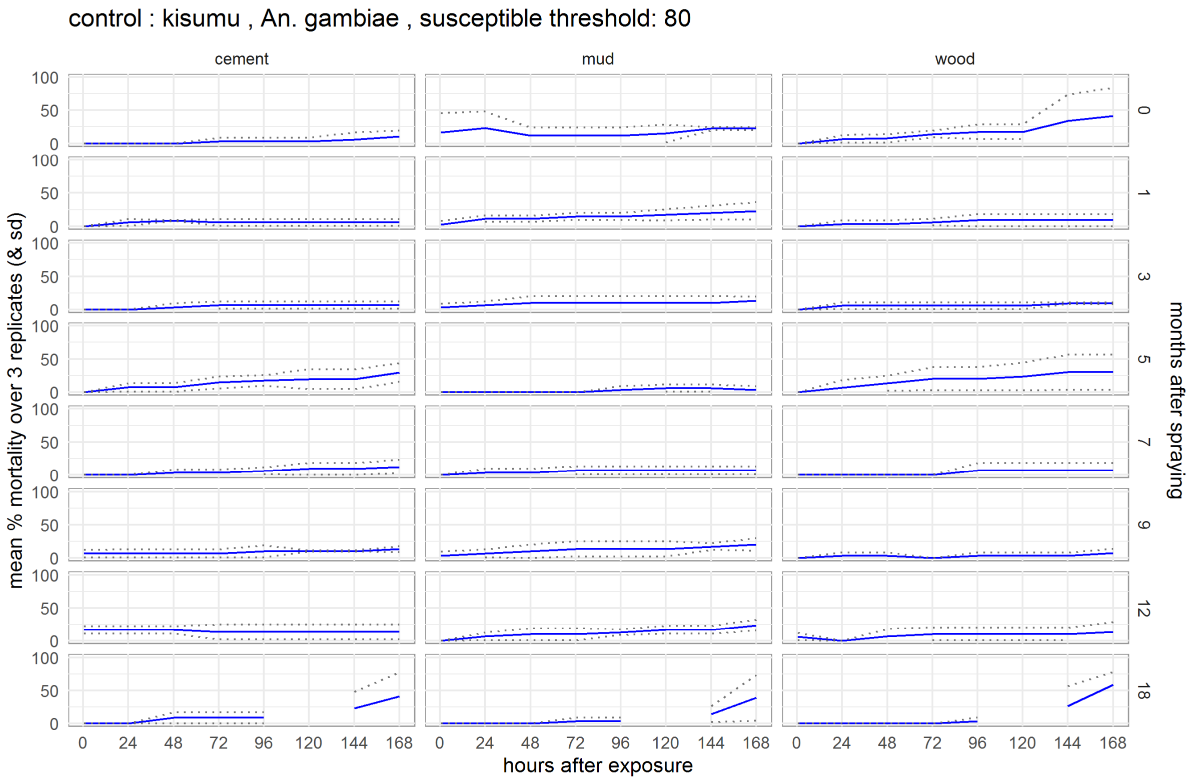

Figure S13. Speed of action of purified water control against insecticide-susceptible *An. gambiae* kisumu. Cumulative mortality is shown by hours post-exposure to treated cement, mud, and wood surfaces, for each month of the experiment. Time to reach the WHO-recommended 80% mortality threshold for an IRS product is marked with a red line

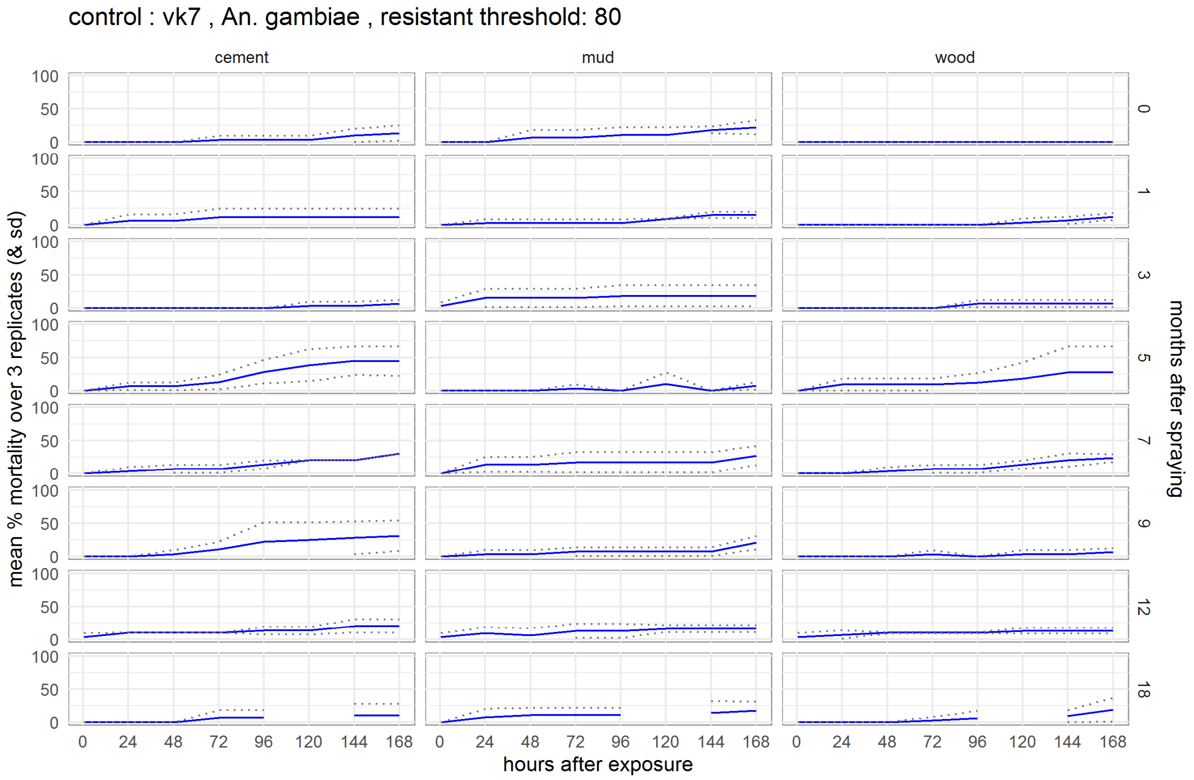

Figure S14. Speed of action of purified water control against insecticide-resistant *An. gambiae* VK7 2014. Cumulative mortality is shown by hours post-exposure to treated cement, mud, and wood surfaces, for each month of the experiment. Time to reach the WHO-recommended 80% mortality threshold for an IRS product is marked with a red line

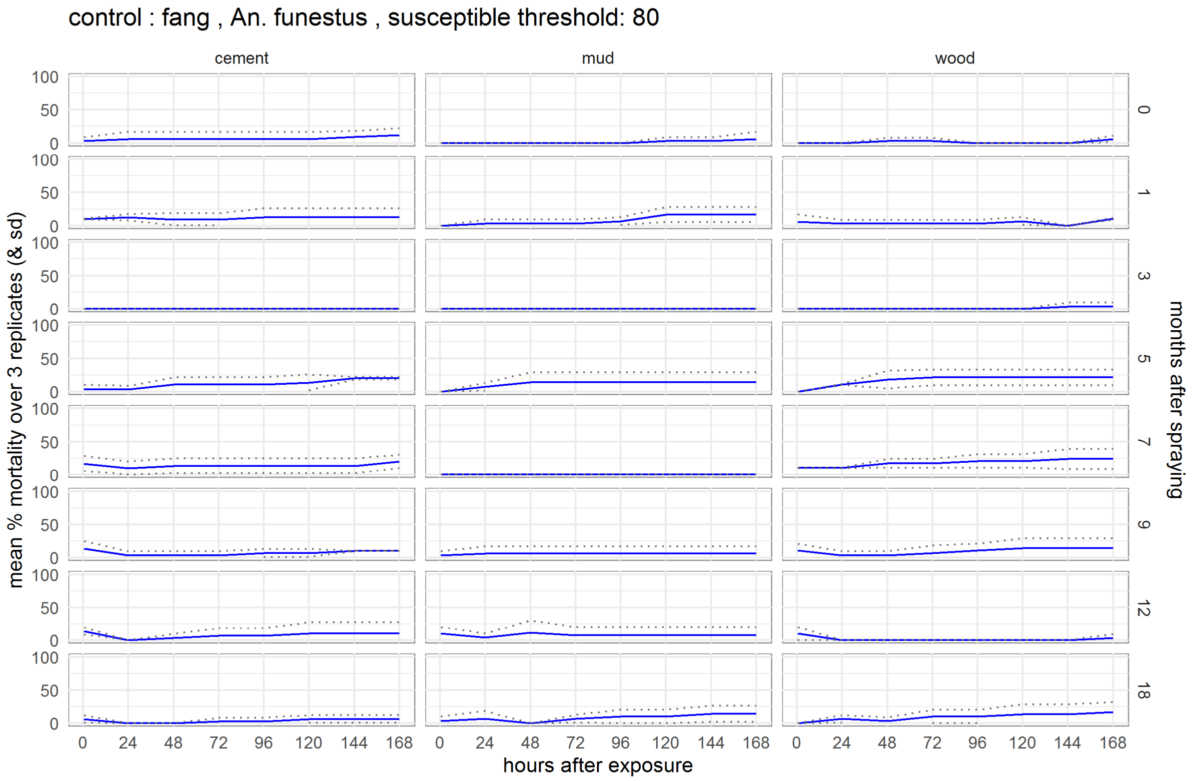

Figure S15. Speed of action of purified water control against insecticide-susceptible *An. funestus* Fang. Cumulative mortality is shown by hours post-exposure to treated cement, mud, and wood surfaces, for each

month of the experiment. Time to reach the WHO-recommended 80% mortality threshold for an IRS product is marked with a red line.

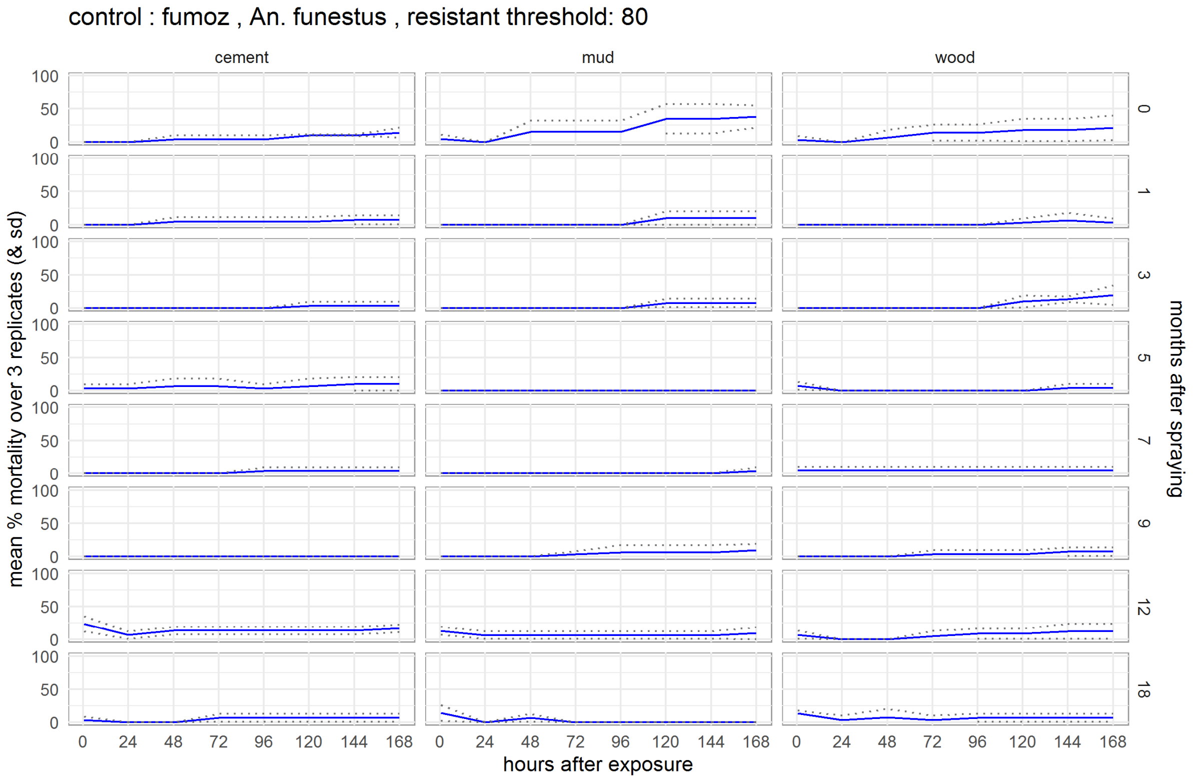

Figure S16. Speed of action of purified water control against insecticide-resistant *An. funestus* FUMOZ-R. Cumulative mortality is shown by hours post-exposure to treated cement, mud, and wood surfaces, for each month of the experiment. Time to reach the WHO-recommended 80% mortality threshold for an IRS product is marked with a red line

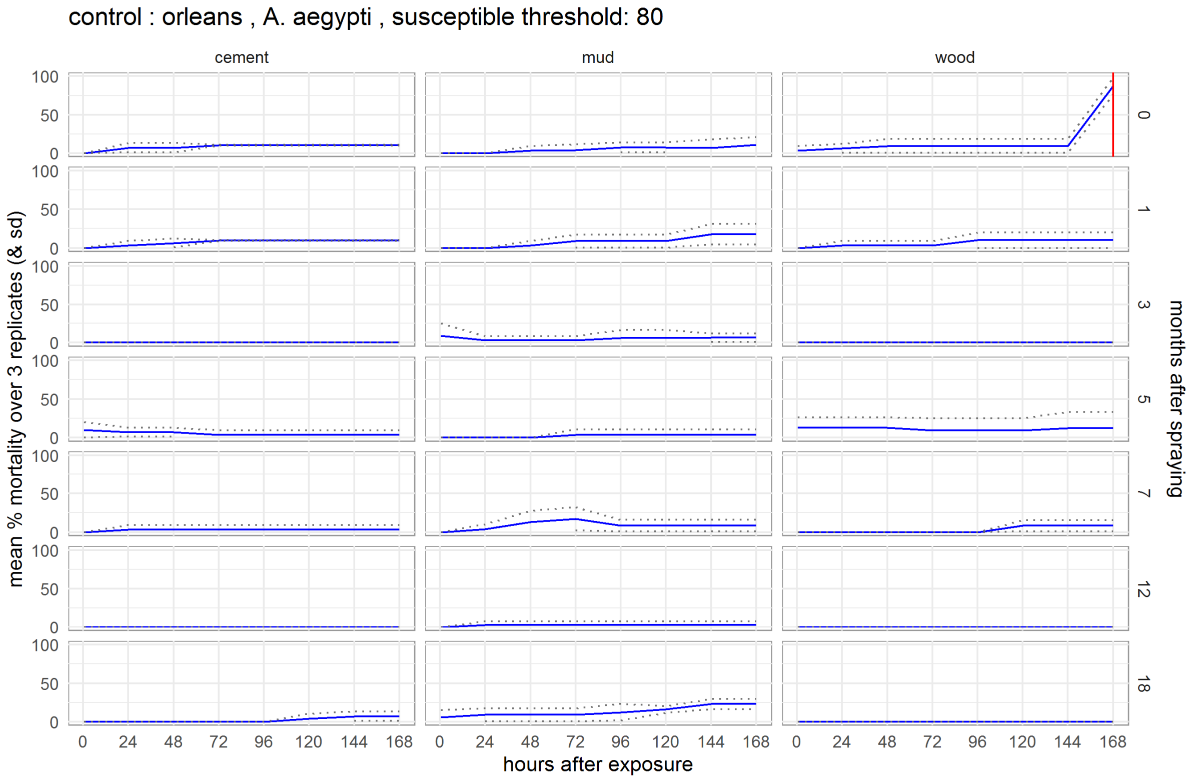

Figure S17. Speed of action of purified water control against insecticide-susceptible *Ae. aegypti* New Orleans Cumulative mortality is shown by hours post-exposure to treated cement, mud, and wood surfaces, for each month of the experiment. Time to reach the WHO-recommended 80% mortality threshold for an IRS product is marked with a red line.

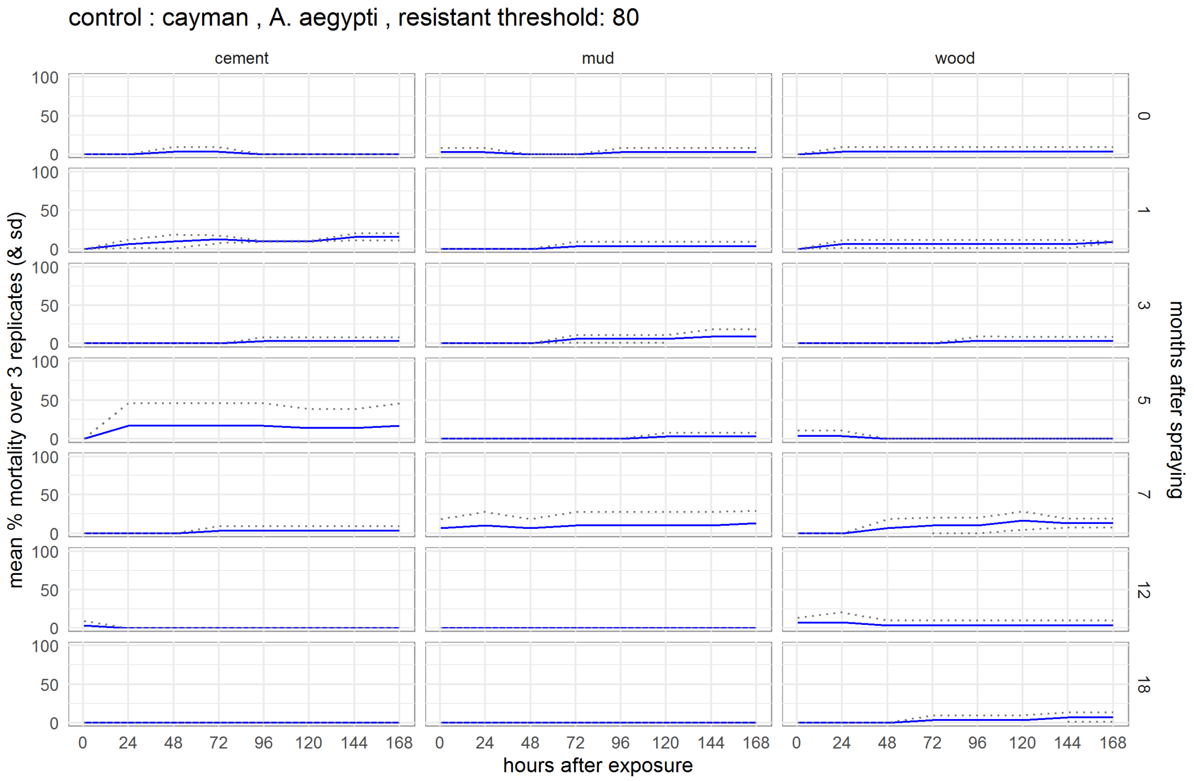

Figure S18. Speed of action of purified water control against insecticide-resistant *Ae. aegypti* Cayman. Cumulative mortality is shown by hours post-exposure to treated cement, mud, and wood surfaces, for each

month of the experiment. Time to reach the WHO-recommended 80% mortality threshold for an IRS product is marked with a red line.

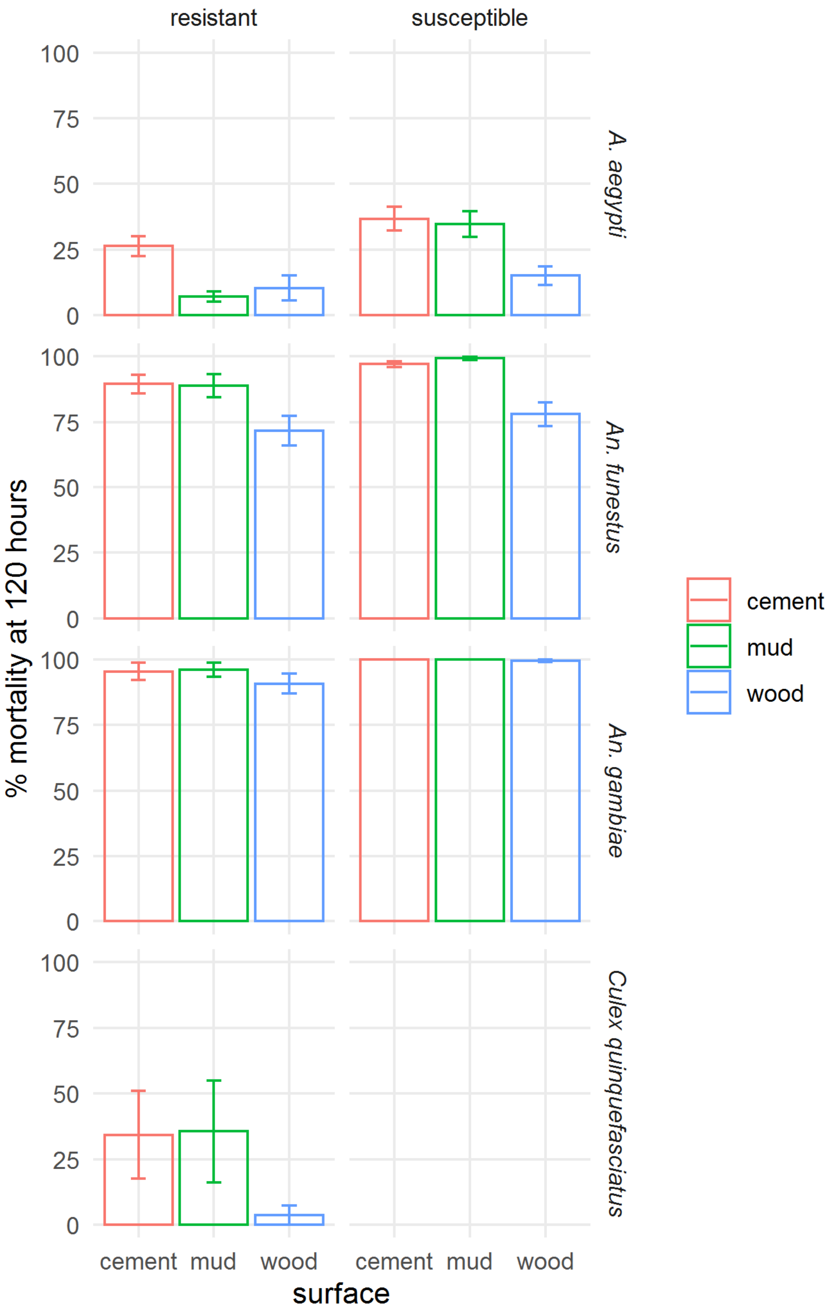

Figure S19. Effect of surface type on efficacy of SumiShield. Mortality of insecticide-susceptible and resistant *An. gambiae*, *An. funestus* and *Ae. aegypti*, and resistant *Cx. quinquefasciatus*. Average 120-hour mortality calculated across all replicate bioassays at all time points (0, 1, 3, 5, 7, 9, 12 and 18 months) for each strain and surface type is shown; error bars represent standard error across 3 replicate assays.

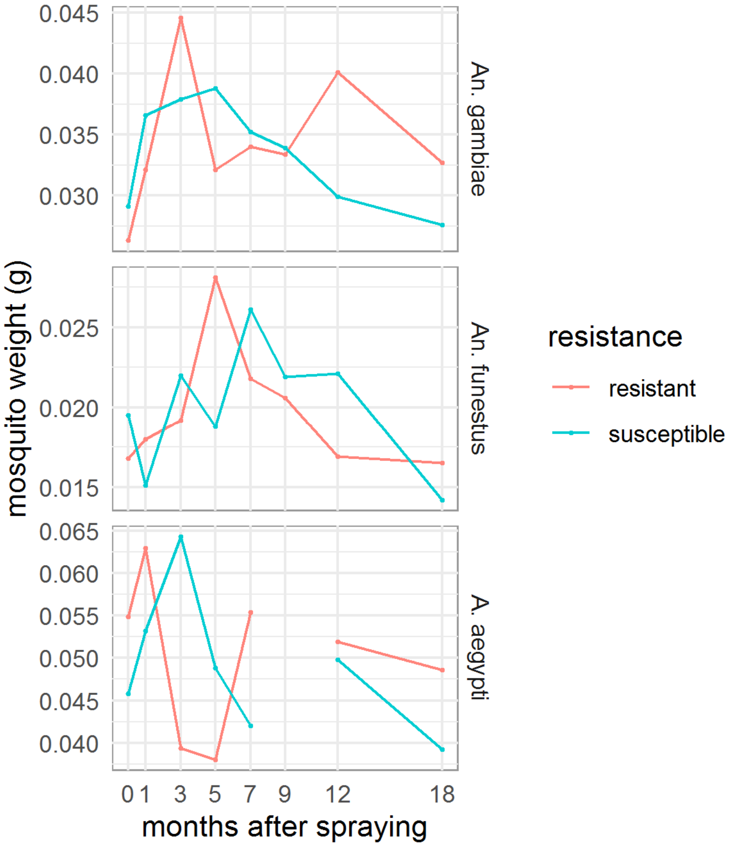

Figure S20. Colony weight in susceptible and resistant *An. gambiae, An. funestus* and *Ae. aegypti* at each time point. A sample of twenty females of each colony was weighed (mosquito weight) by anaesthetising them with carbon dioxide; these mosquitoes were not included in the mosquitoes used for testing.
