# Supplementary figures and images for: The Residual Efficacy of SumiShield™ 50WG and K-Othrine® WG250 IRS Formulations Applied to Different Building Materials against Anopheles and Aedes Mosquitoes
Source: Insects. 2022 Jan 20;13(2):112. doi: 10.3390/insects13020112 (PMC8877416; doi:10.3390/insects13020112)

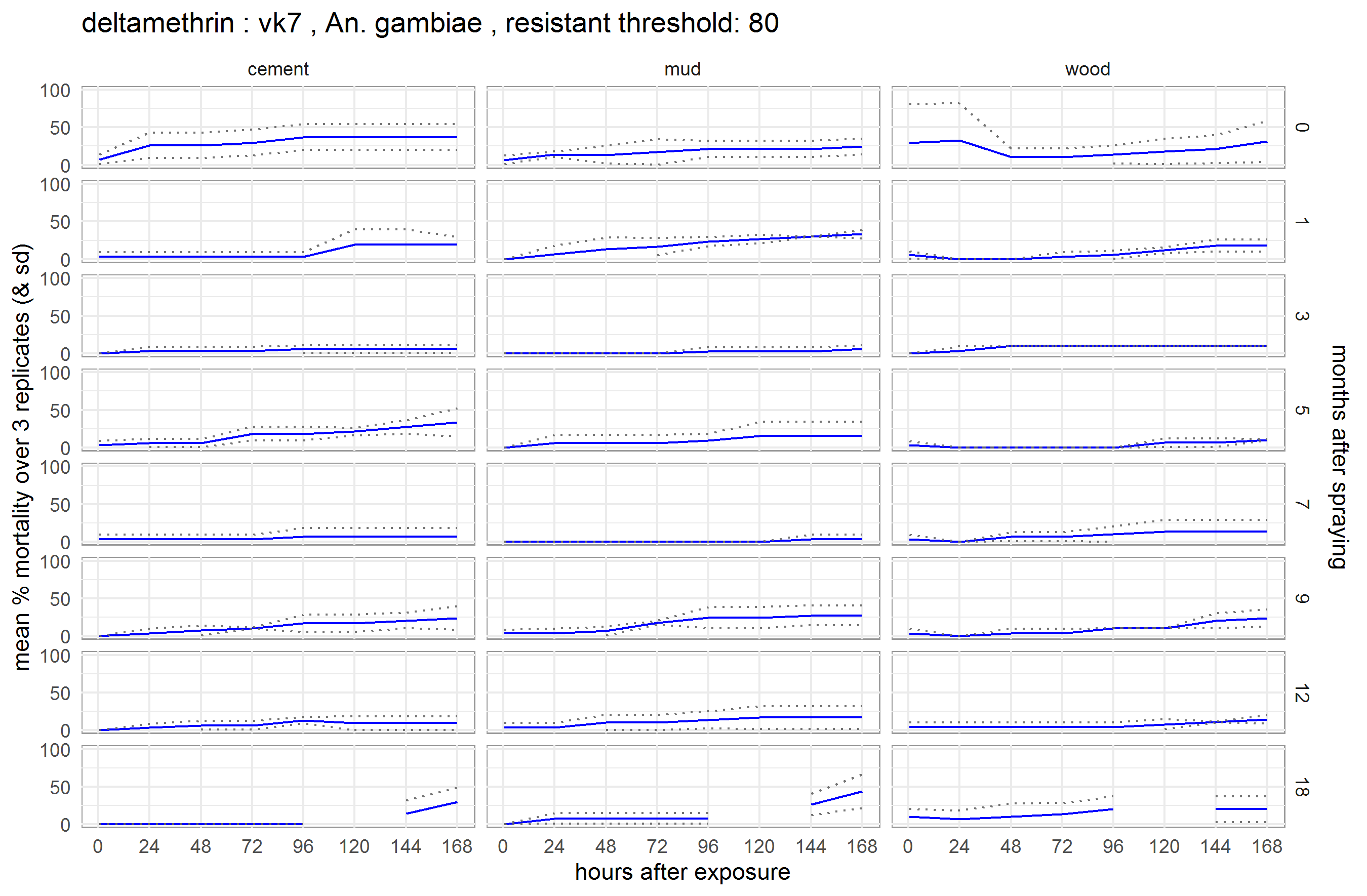

Supplement: Supplementary file 1 [file insects-13-00112-s001.zip › insects-1516983-SI/Supplementary Material/Figure S10_mort_by_time_after_exp80-8.tiff]

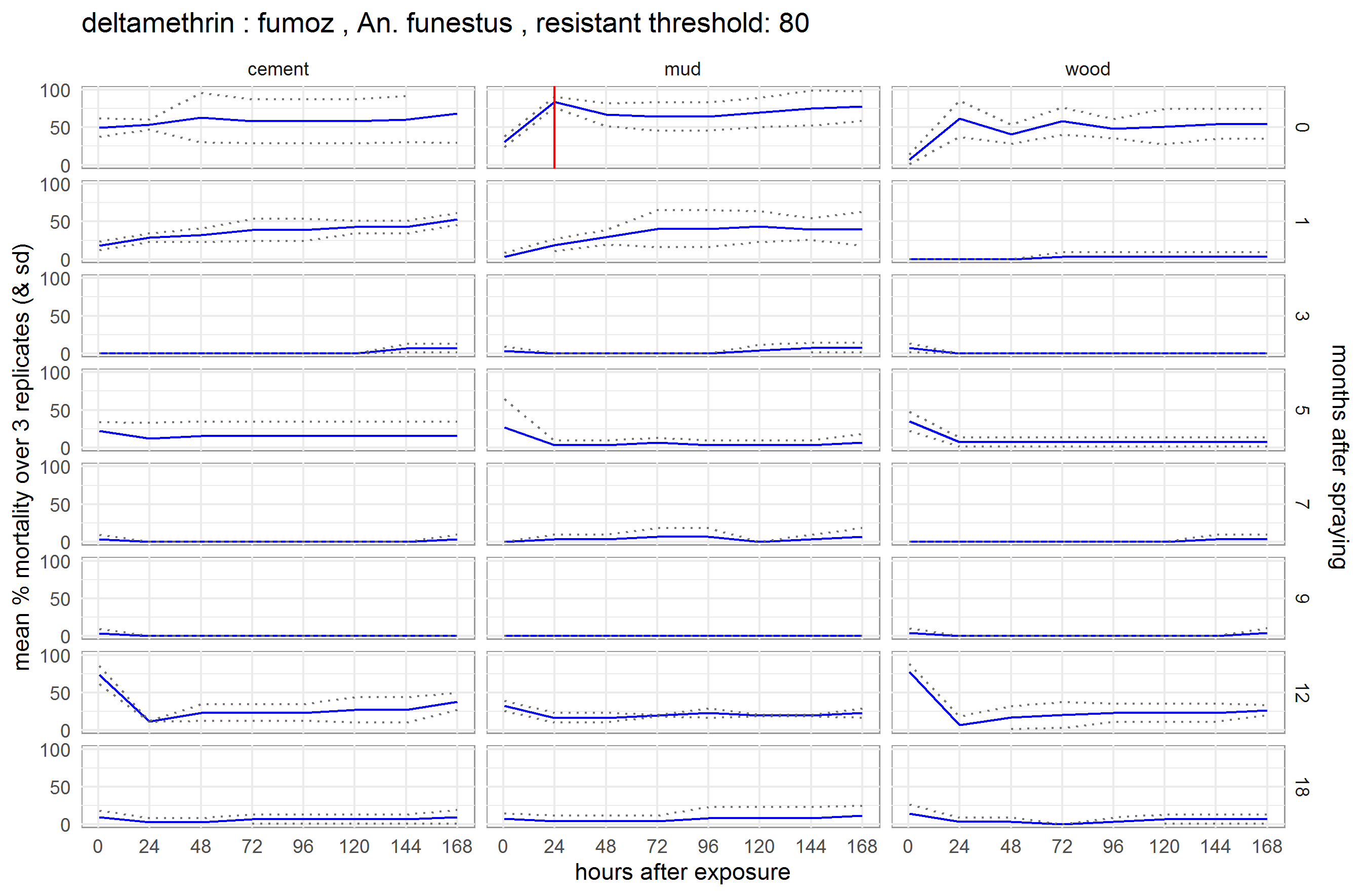

Supplement: Supplementary file 1 [file insects-13-00112-s001.zip › insects-1516983-SI/Supplementary Material/Figure S11_mort_by_time_after_exp80-10.tiff]

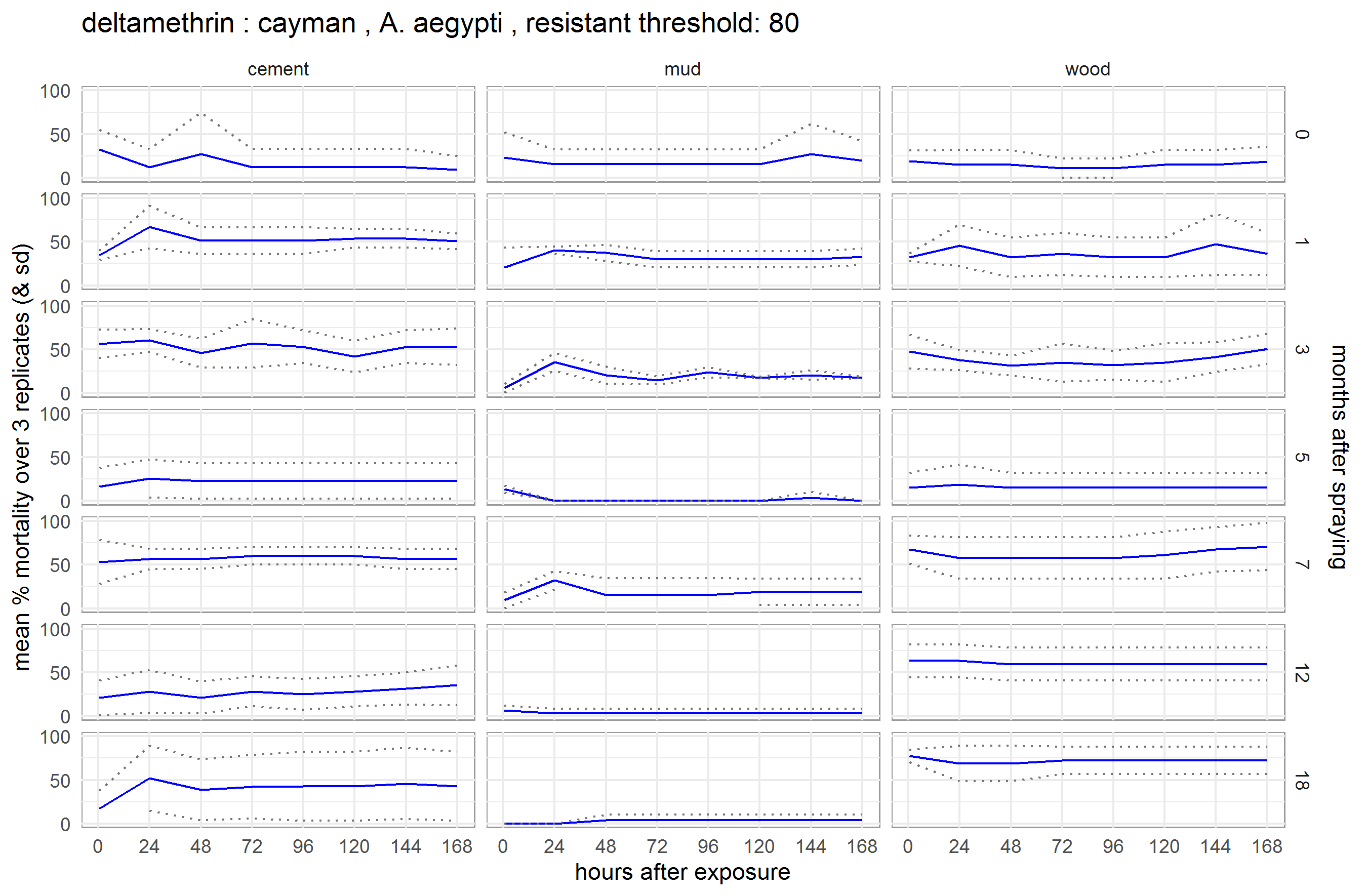

Supplement: Supplementary file 1 [file insects-13-00112-s001.zip › insects-1516983-SI/Supplementary Material/Figure S12_mort_by_time_after_exp80-12.tiff]

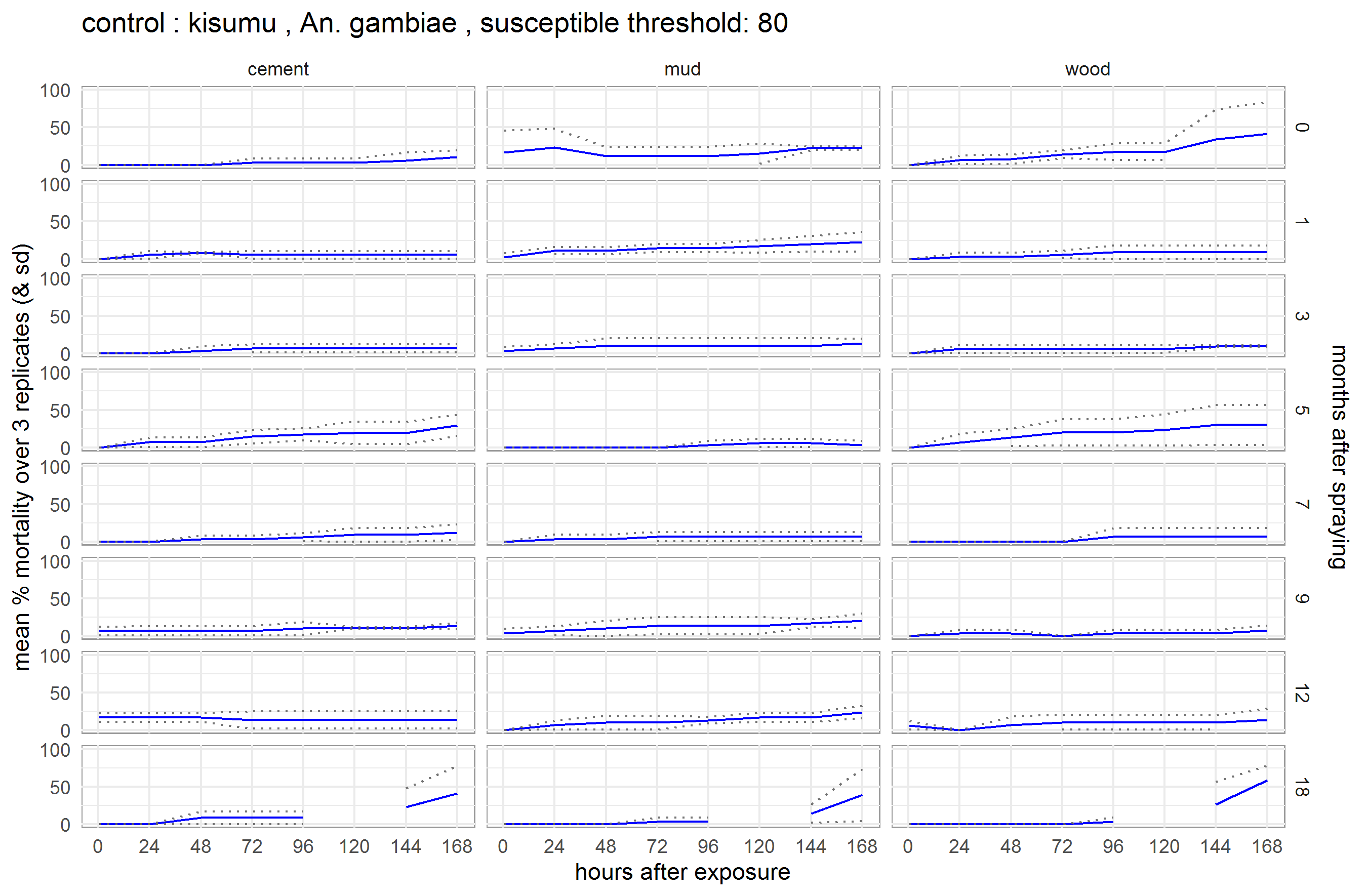

Supplement: Supplementary file 1 [file insects-13-00112-s001.zip › insects-1516983-SI/Supplementary Material/Figure S13_mort_by_time_after_exp80-13.tiff]

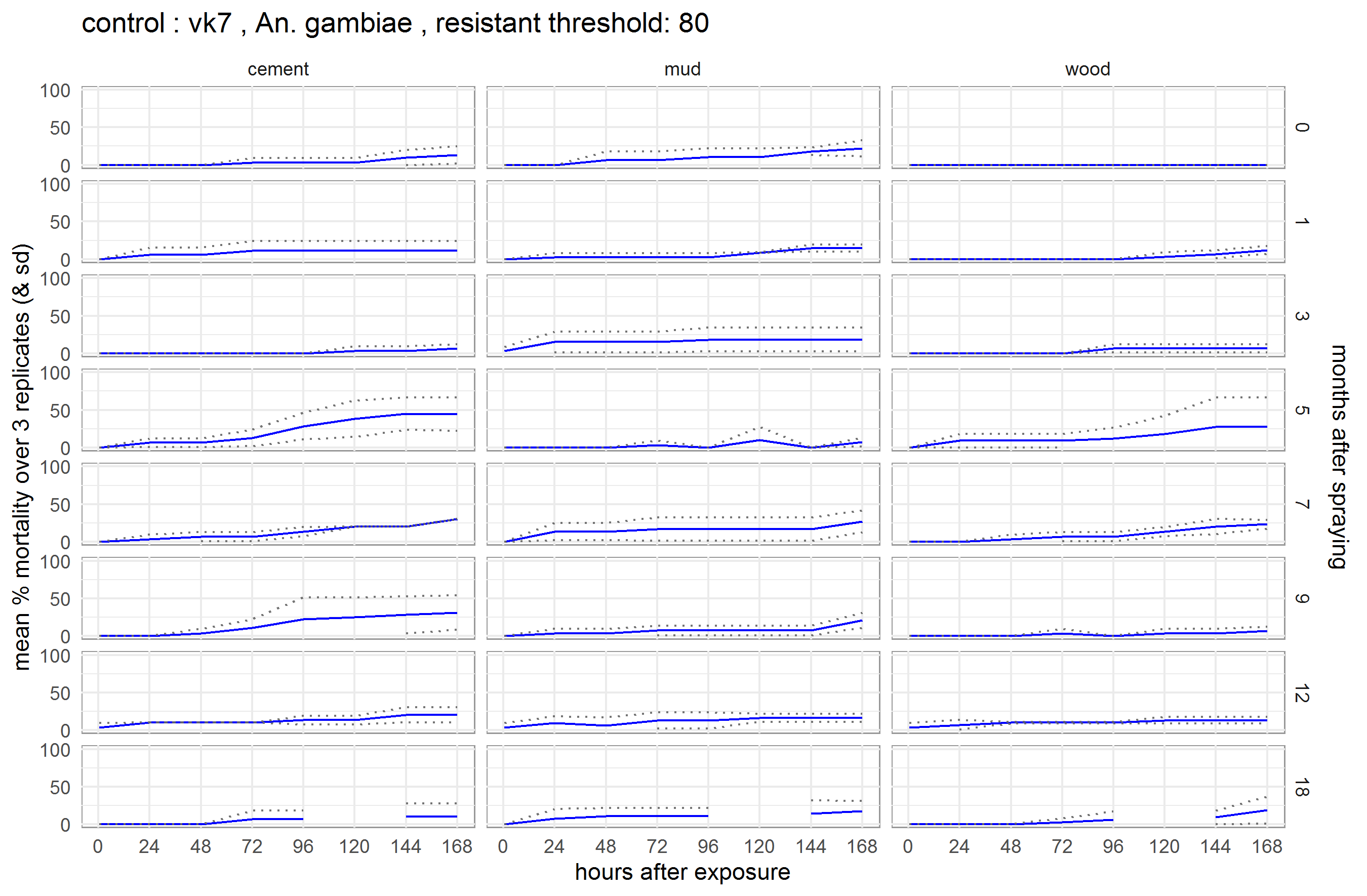

Supplement: Supplementary file 1 [file insects-13-00112-s001.zip › insects-1516983-SI/Supplementary Material/Figure S14_mort_by_time_after_exp80-14.tiff]

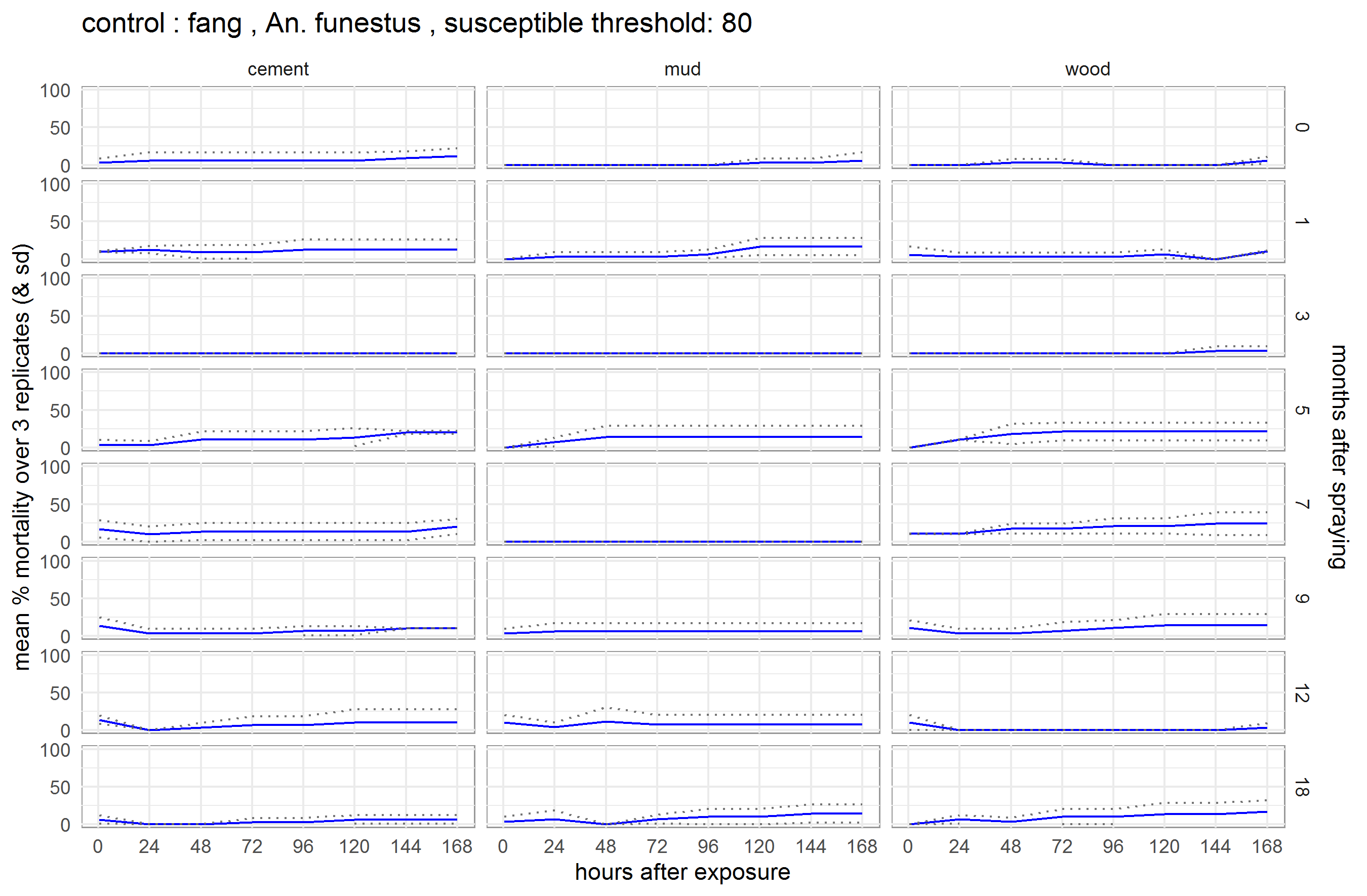

Supplement: Supplementary file 1 [file insects-13-00112-s001.zip › insects-1516983-SI/Supplementary Material/Figure S15_mort_by_time_after_exp80-15.tiff]

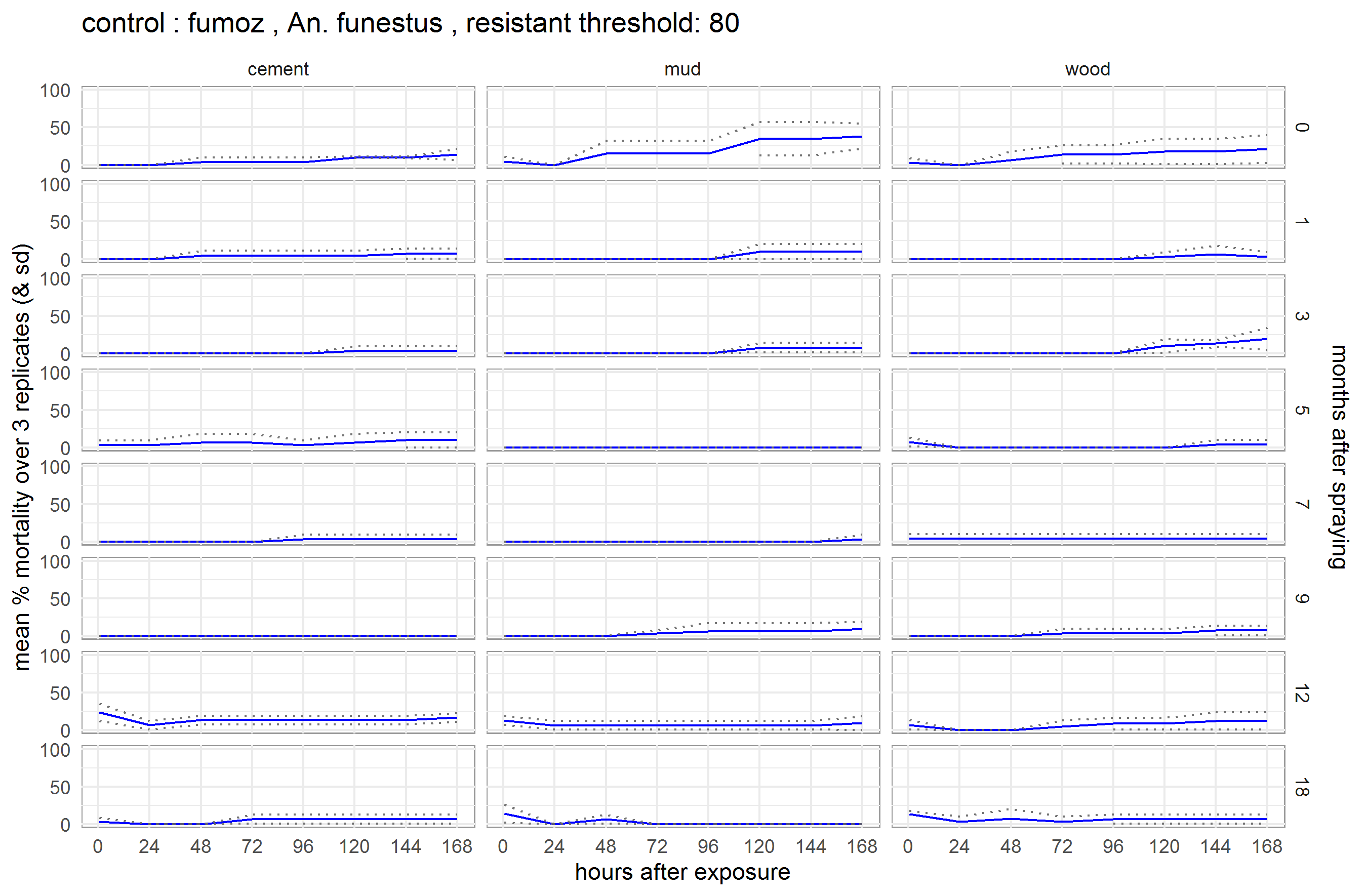

Supplement: Supplementary file 1 [file insects-13-00112-s001.zip › insects-1516983-SI/Supplementary Material/Figure S16_mort_by_time_after_exp80-16.tiff]

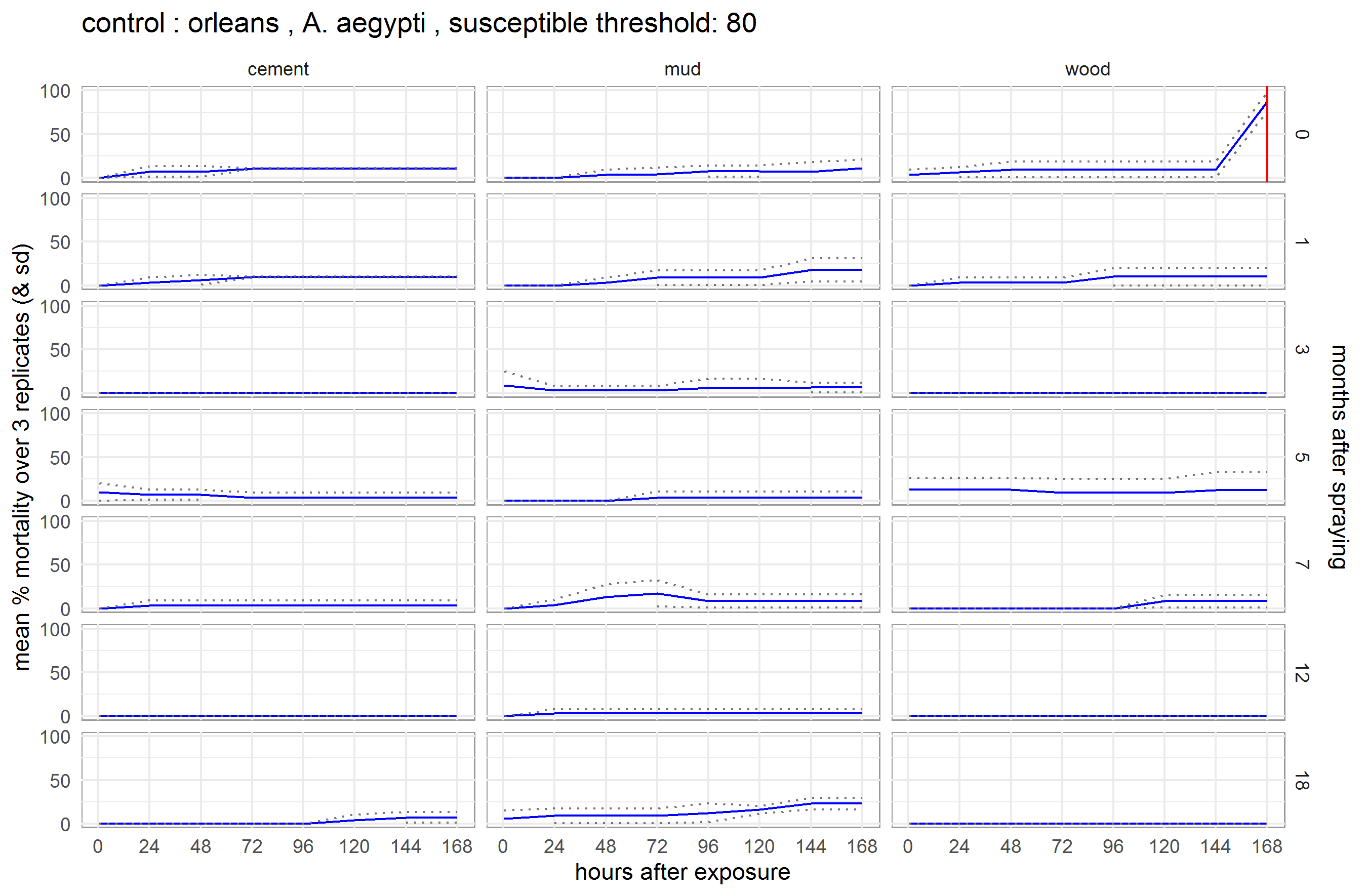

Supplement: Supplementary file 1 [file insects-13-00112-s001.zip › insects-1516983-SI/Supplementary Material/Figure S17_mort_by_time_after_exp80-17.tiff]

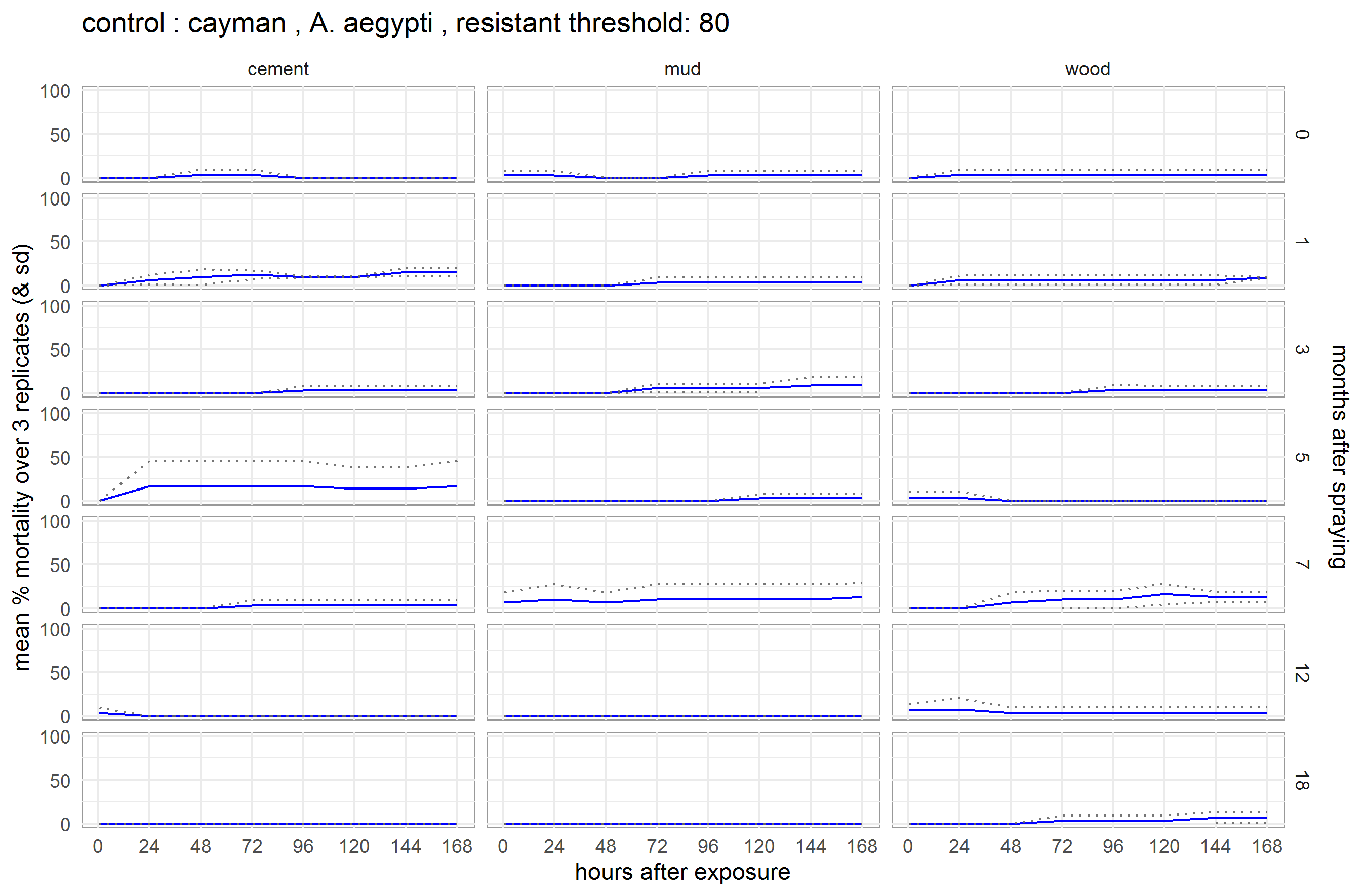

Supplement: Supplementary file 1 [file insects-13-00112-s001.zip › insects-1516983-SI/Supplementary Material/Figure S18_mort_by_time_after_exp80-18.tiff]

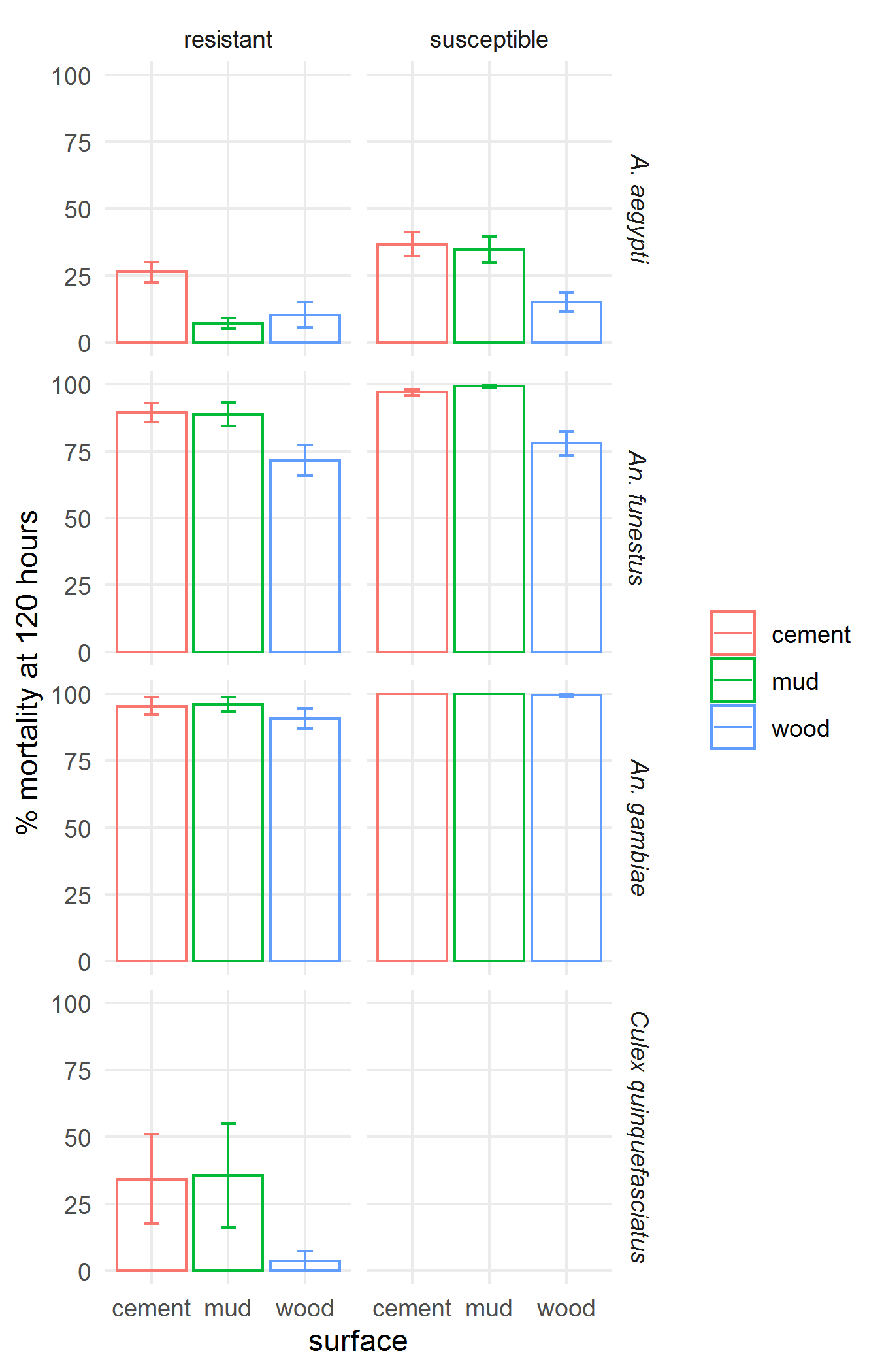

Supplement: Supplementary file 1 [file insects-13-00112-s001.zip › insects-1516983-SI/Supplementary Material/Figure S19_fig5-mean-sumi-mort120-surface-resistance-species-1.tiff]

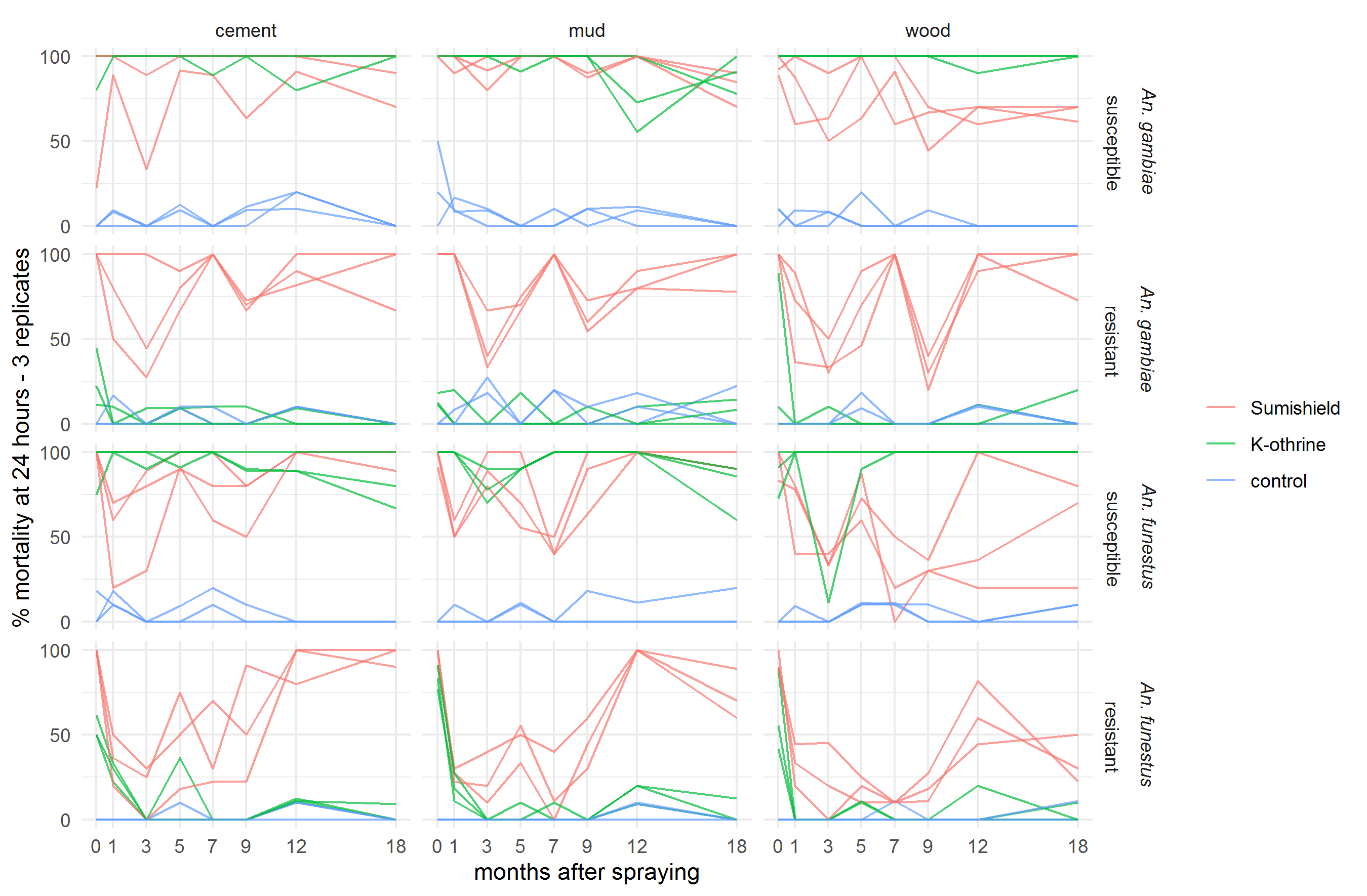

Supplement: Supplementary file 1 [file insects-13-00112-s001.zip › insects-1516983-SI/Supplementary Material/Figure S1_fig2-sumi-delt-mort24-months-1.tiff]

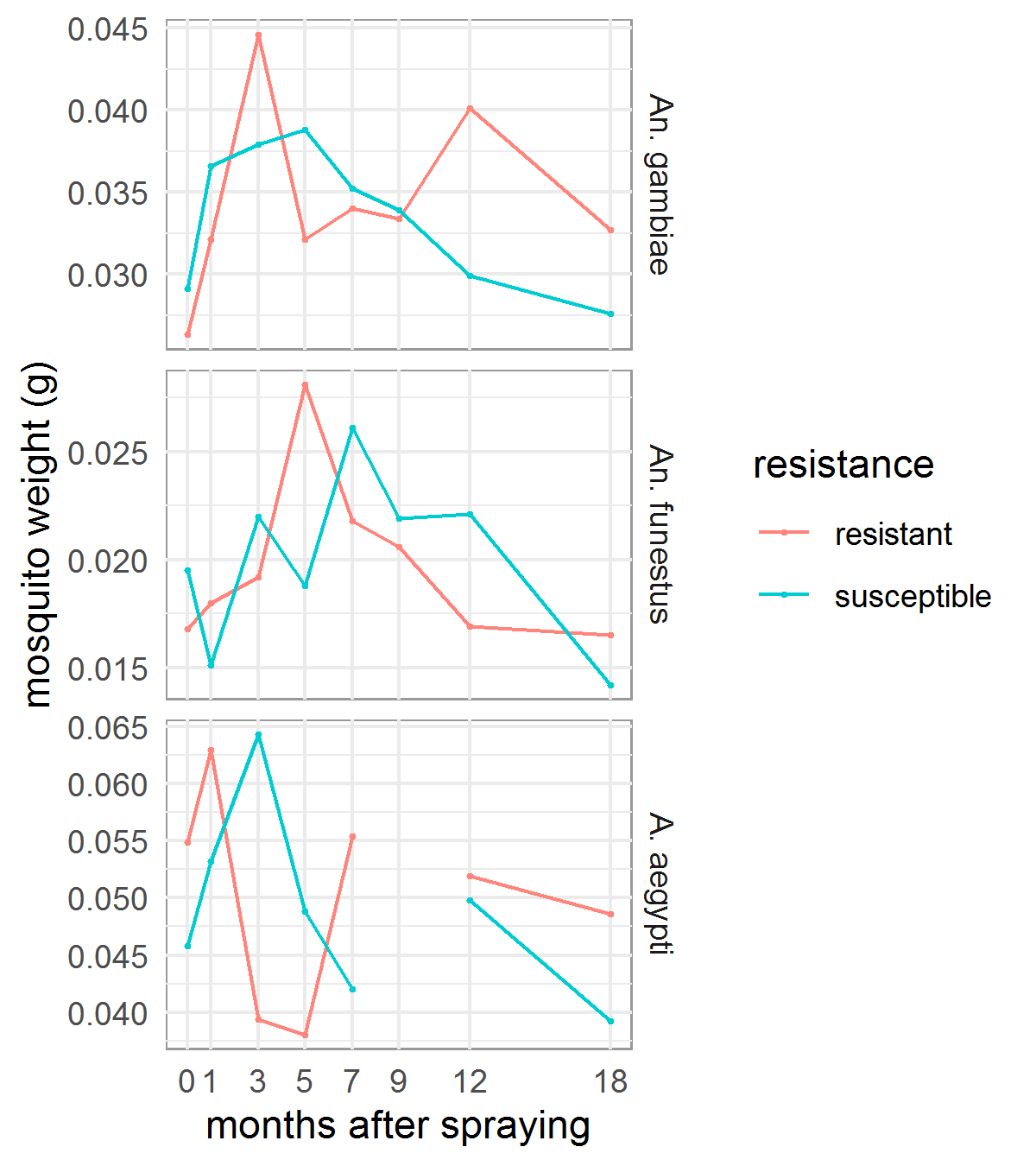

Supplement: Supplementary file 1 [file insects-13-00112-s001.zip › insects-1516983-SI/Supplementary Material/Figure S20_figS21-weights-1.tiff]

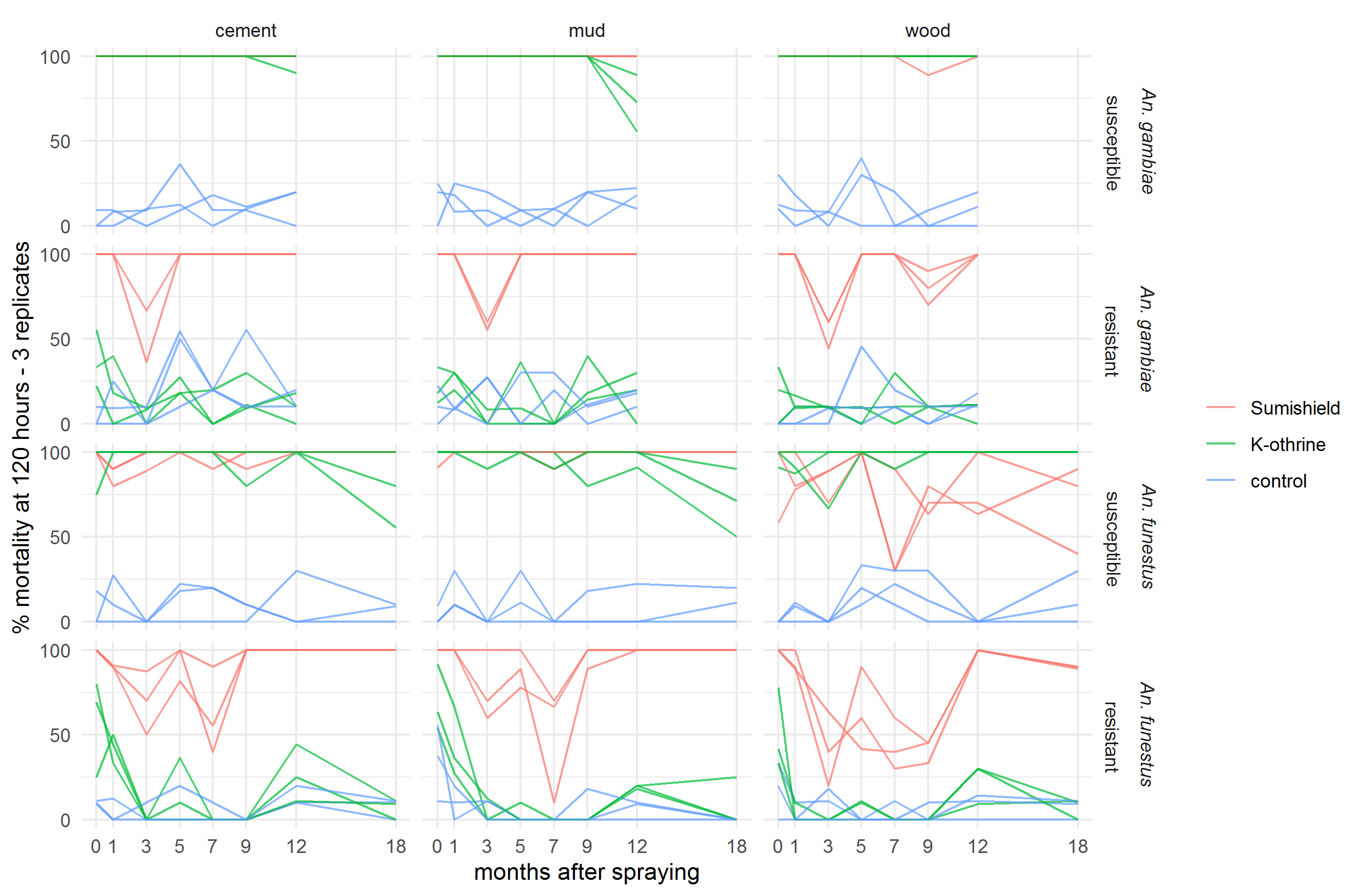

Supplement: Supplementary file 1 [file insects-13-00112-s001.zip › insects-1516983-SI/Supplementary Material/Figure S2_fig2-sumi-delt-mort120-months-1.tiff]

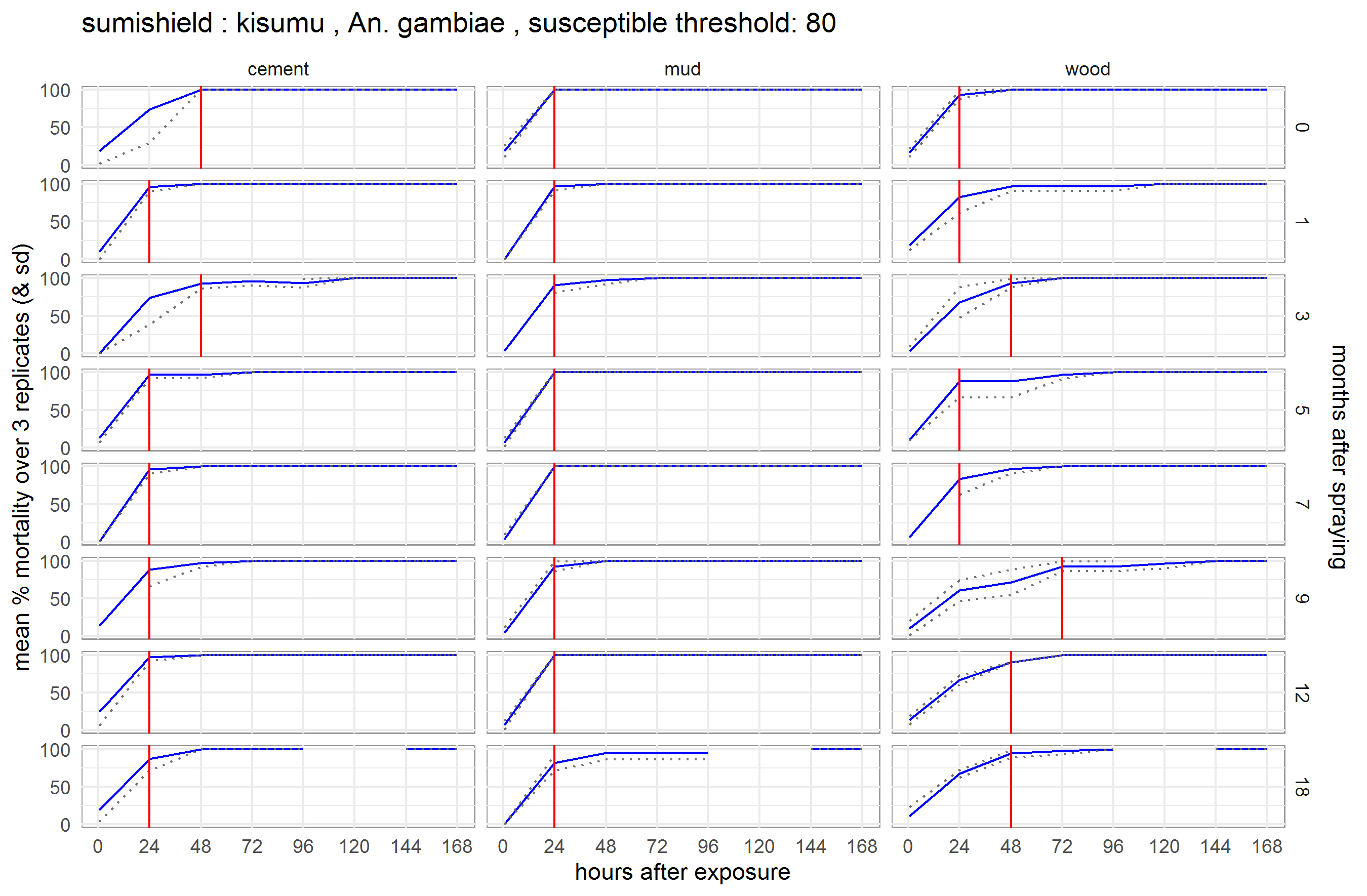

Supplement: Supplementary file 1 [file insects-13-00112-s001.zip › insects-1516983-SI/Supplementary Material/Figure S3_mort_by_time_after_exp80-1.tiff]

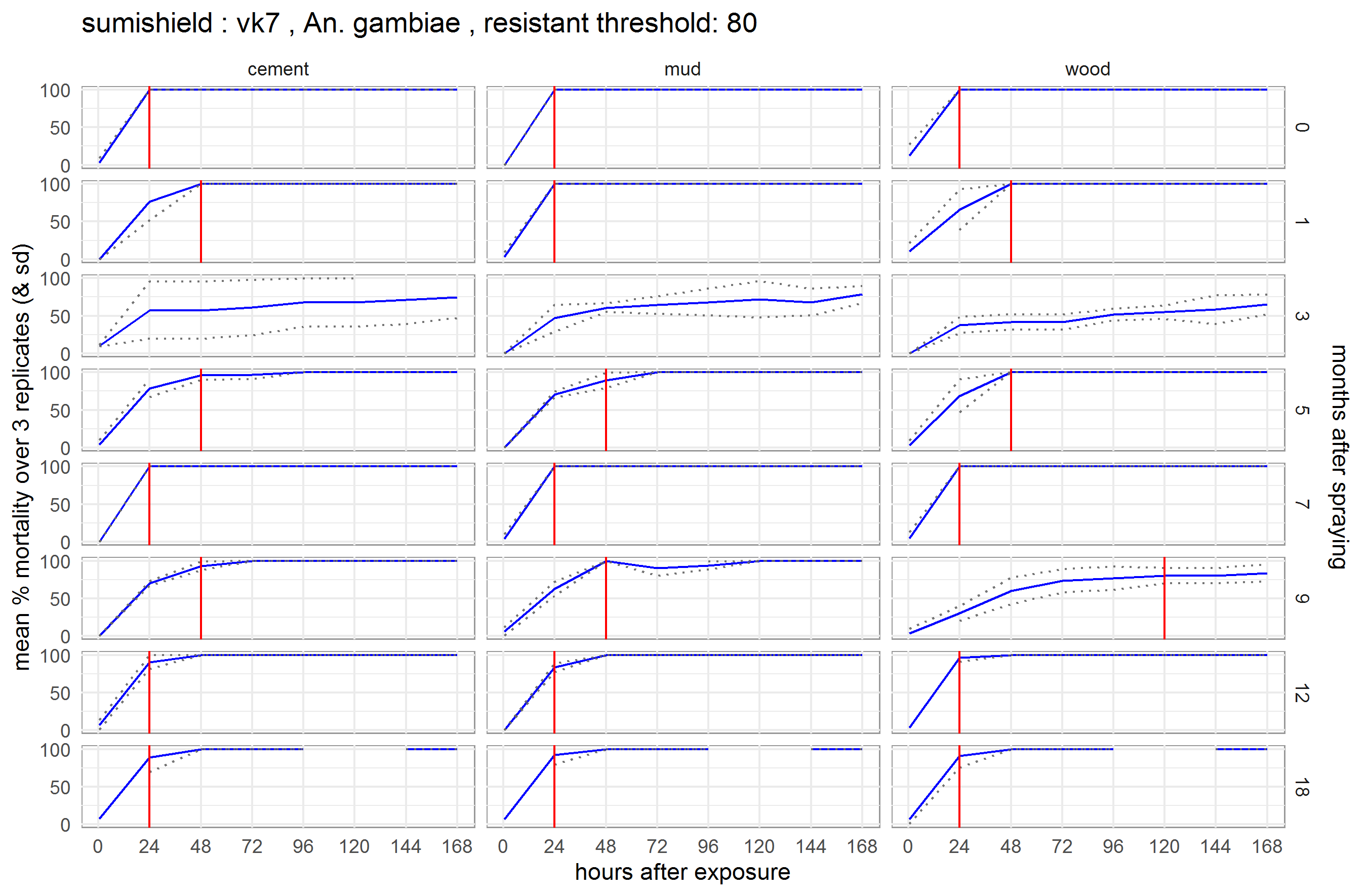

Supplement: Supplementary file 1 [file insects-13-00112-s001.zip › insects-1516983-SI/Supplementary Material/Figure S4_mort_by_time_after_exp80-2.tiff]

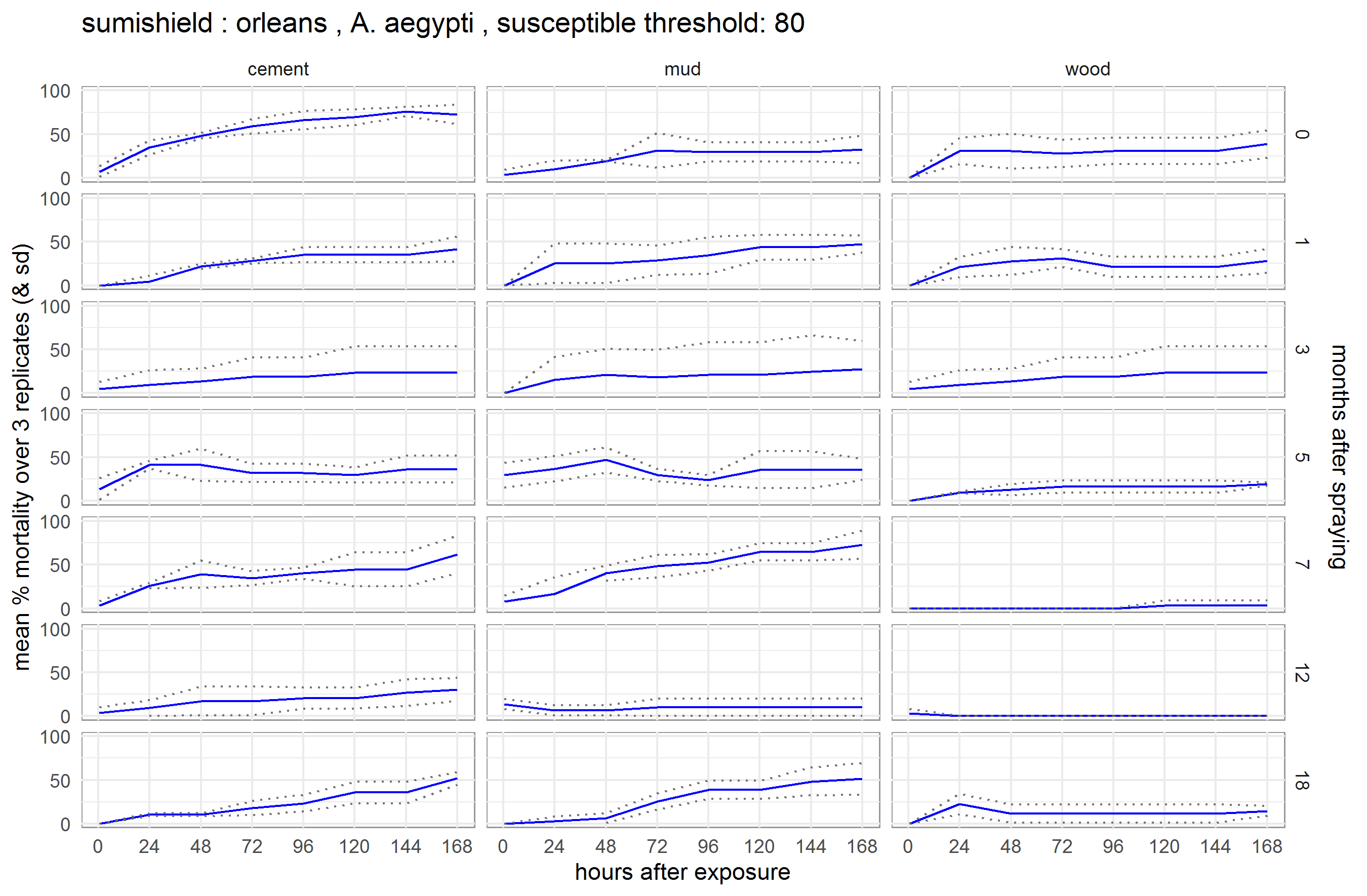

Supplement: Supplementary file 1 [file insects-13-00112-s001.zip › insects-1516983-SI/Supplementary Material/Figure S5_mort_by_time_after_exp80-5.tiff]

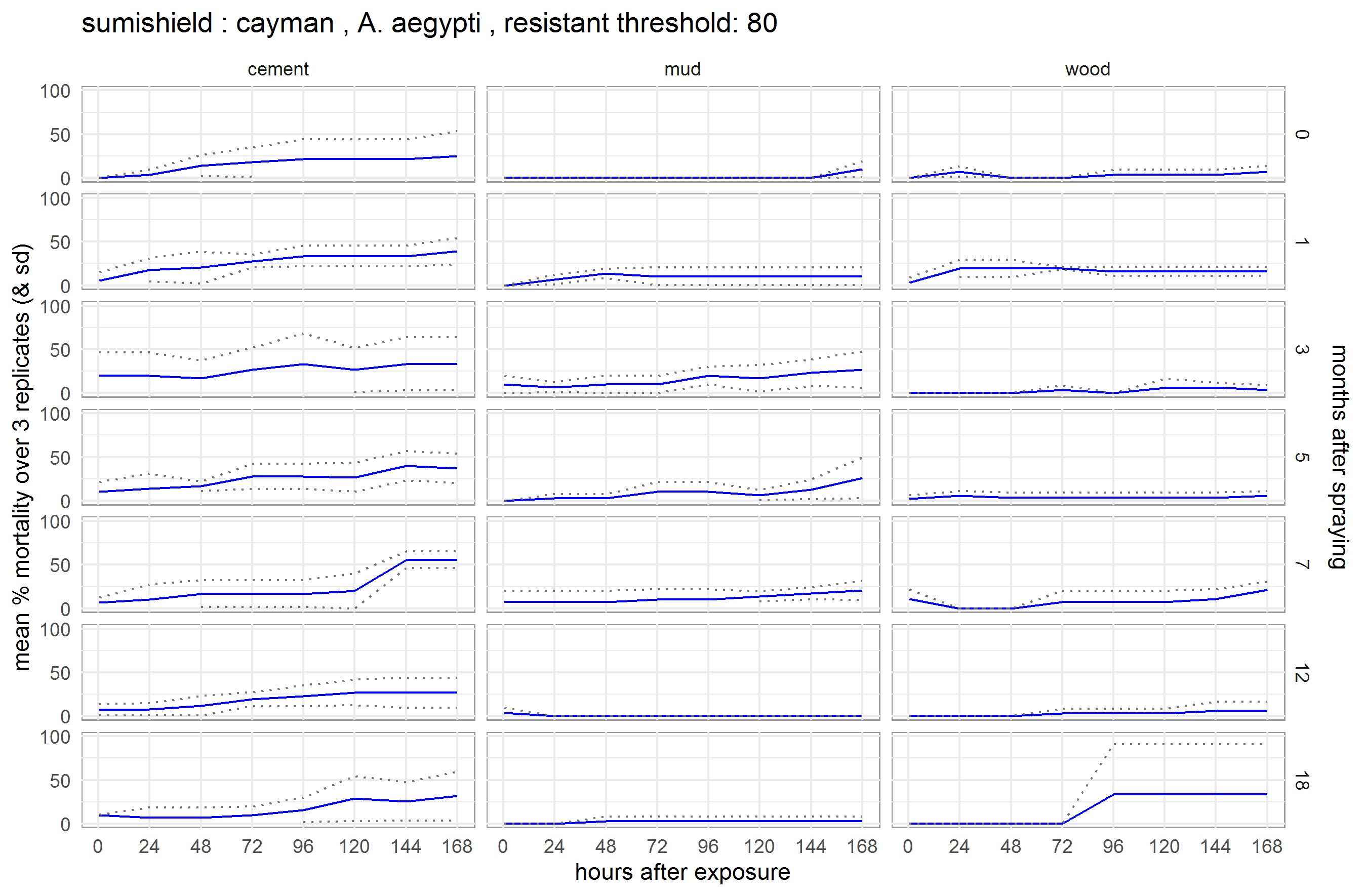

Supplement: Supplementary file 1 [file insects-13-00112-s001.zip › insects-1516983-SI/Supplementary Material/Figure S6_mort_by_time_after_exp80-6.tiff]

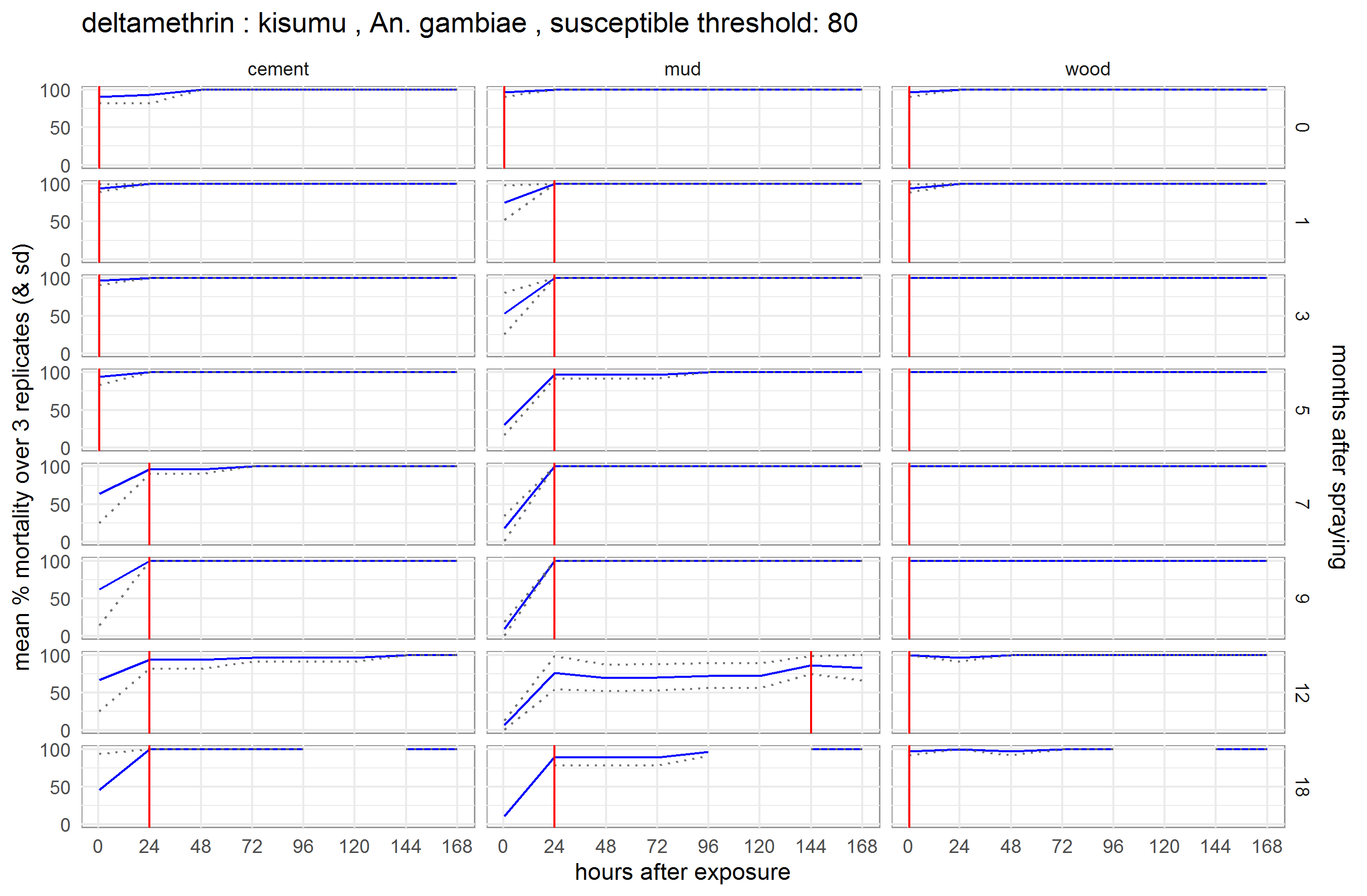

Supplement: Supplementary file 1 [file insects-13-00112-s001.zip › insects-1516983-SI/Supplementary Material/Figure S7_mort_by_time_after_exp80-7.tiff]

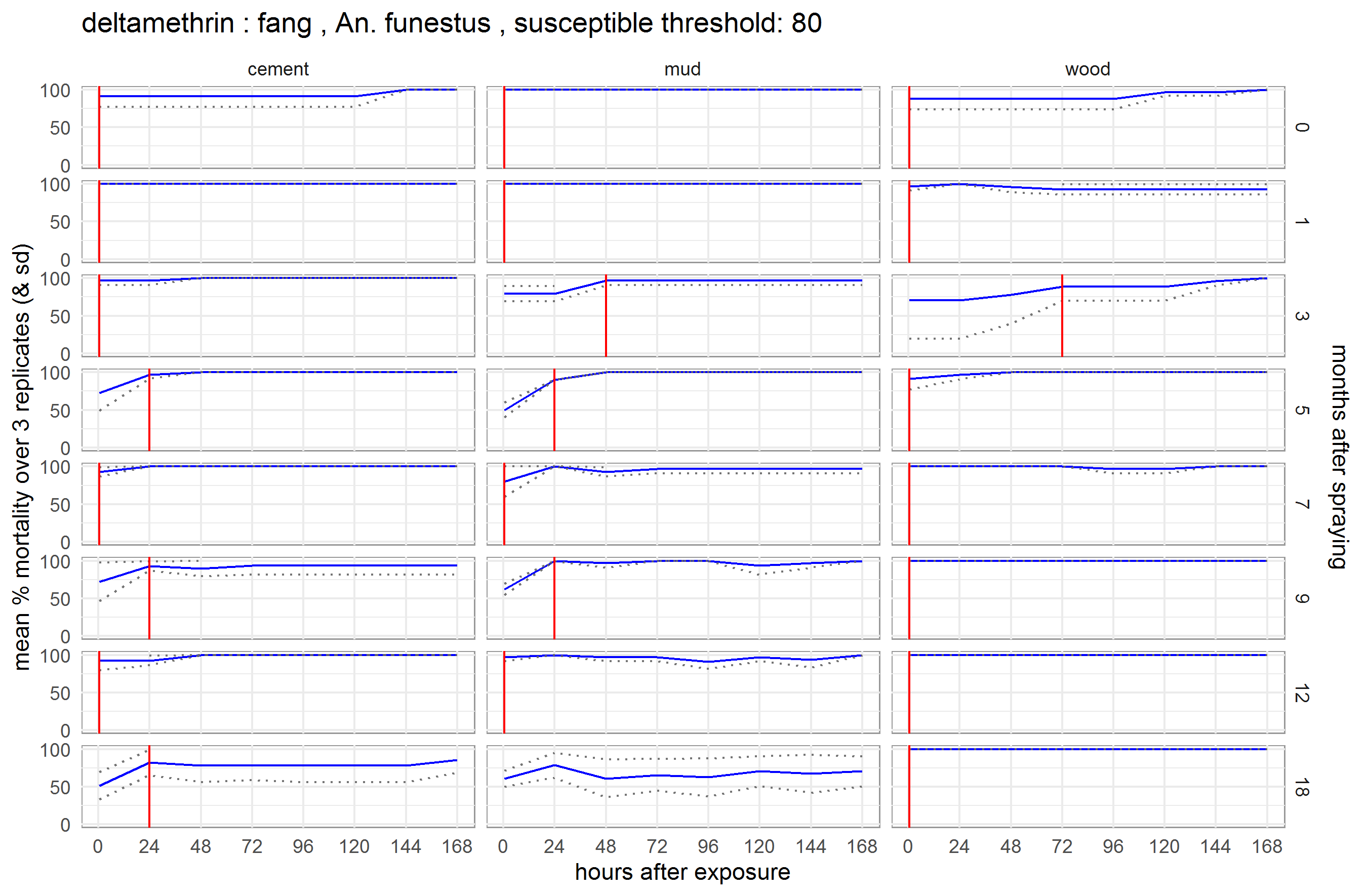

Supplement: Supplementary file 1 [file insects-13-00112-s001.zip › insects-1516983-SI/Supplementary Material/Figure S8_mort_by_time_after_exp80-9.tiff]

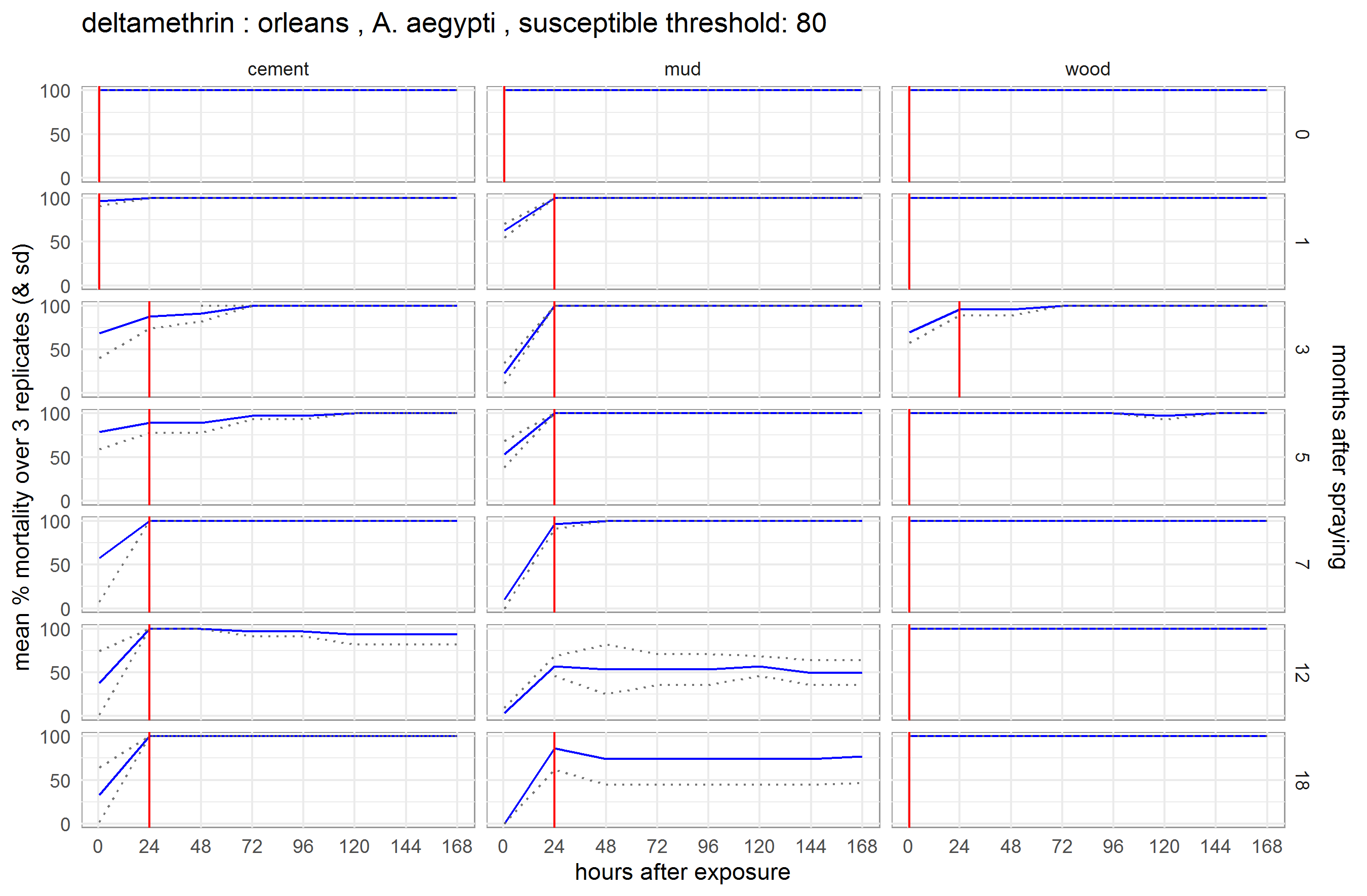

Supplement: Supplementary file 1 [file insects-13-00112-s001.zip › insects-1516983-SI/Supplementary Material/Figure S9_mort_by_time_after_exp80-11.tiff]
